# Fifteen-Year Nationwide Trend in Antiplatelet Treatment among Drug-Eluting Stent Recipients in Korea: Many Patients Receive Very Prolonged Dual-Antiplatelet Treatment, and Newer Drugs Are Replacing the Older Ones

**DOI:** 10.3390/jcm12072675

**Published:** 2023-04-03

**Authors:** Sunwon Kim, Jong-Seok Lee, Jungkuk Lee, Yong-Hyun Kim, Jin-Seok Kim, Sang-Yup Lim, Seong Hwan Kim, Jeong-Cheon Ahn, Woo-Hyuk Song

**Affiliations:** 1 Cardiovascular Center, Korea University Ansan Hospital, Ansan-si 15355, Republic of Korea; 2 Hanmi Pharmaceuticals, Songpa-gu, Seoul 05545, Republic of Korea

**Keywords:** antiplatelet treatment, clopidogrel, drug-eluting stent, dual-antiplatelet treatment, ticagrelor

## Abstract

Drug-eluting stent (DES) recipients require 6–12 months of dual antiplatelet treatment (DAPT) and long-term aspirin mono-antiplatelet treatment (MAPT). Given the diversity of contemporary antiplatelet agents, antiplatelet treatment (APT) selection is becoming more complicated. We evaluated 15-year APT trends based on nationwide prescription data of 79,654 patients who underwent percutaneous coronary intervention (PCI) using DESs from 2002 to 2018 in Korea. DAPT (80.7%) was the most preferred initial APT post-PCI. Many DES recipients received prolonged DAPT (post-PCI 3 years: 41.0%; 10 years: 27.7%). There was a noticeable delay in DAPT-to-MAPT conversion from the mid to late 2000s (after the late-stent thrombosis concerns of first-generation DESs raised); the conversion after that was similar during the 2010s, occurring most robustly at 12–18 months post-PCI. Clopidogrel had long and increasingly been used for MAPT, surpassing aspirin. The recent increase in newer P2Y12 inhibitor prescriptions was noted. The patients treated with newer P2Y12 inhibitors were more likely younger men and presented with acute myocardial infarction. Real-world APT is evolving, and guideline–practice gaps exist. Further studies exploring the impact of diverse APT strategies on patient outcomes are expected to provide insights into optimal APT that can sophisticatedly balance the ischemic and bleeding risks.

## 1. Introduction

The introduction of metallic stents was a breakthrough in the field of interventional cardiology. In particular, the development of stents coated with antiproliferative drugs [i.e., drug-eluting stents (DESs)] reflects technological improvements for achieving better efficacy and safety [1,2]. DESs are currently the most widely used stents in the contemporary percutaneous coronary intervention (PCI) era, accounting for more than 90% of stents used in the Korea [3]. Despite their efficacy in preventing acute restenosis compared to bare metal stents, accumulated evidence demonstrated delayed arterial healing, late neointimal growth, and development of neo-atherosclerosis associated with DES [4], which necessitate lifelong antiplatelet treatment (APT). Conversely, given that platelet inhibition increases the risk of bleeding, clinicians must decide on individualized APT strategies to achieve the appropriate balance between the ischemic and bleeding risks [5].

Guidelines commonly recommend 6–12 months of dual antiplatelet treatment (DAPT) post-PCI and lifelong aspirin mono-antiplatelet treatment (MAPT) after that, with clopidogrel serving as an alternative in particular cases of aspirin intolerance [6,7]. However, clinicians frequently encounter DES recipients on prolonged DAPT or long-term clopidogrel treatment. A recent randomized clinical trial (RCT) demonstrated the superiority of long-term clopidogrel MAPT over aspirin treatment among DES recipients who had completed post-PCI DAPT uneventfully, challenging aspirin’s role in chronic maintenance APT [8]. Further, with newer P2Y12 inhibitors (P2Y12is), such as prasugrel and ticagrelor, APT selection in this patient population is becoming more complicated [9,10]. Several studies have investigated real-world APT in DES recipients; however, the results were based on small population numbers, short observation periods, and observations confined to a specific disease category or antiplatelet agent and are thus limited to providing a comprehensive understanding [3,11,12]. Based on a nationwide database, this study aimed to investigate the patterns and temporal changes in APT after PCI using DES during the past 15 years, including principal antiplatelet agents commonly used in contemporary practice.

## 2. Materials and Methods

### 2.1. Study Population and Data Sources

Korea has a universal national health insurance system that covers the entire Korean population (approximately 52 million people in 2019). The system provides data regarding nationwide medical claims, sociodemographic information, healthcare utilization, and healthcare provider information. However, the complexity and volume of the data make it difficult for researchers to access and analyze efficiently, and security policies restrict full access. To address these issues, Health Insurance Review and Assessment Service provides researchers with an anonymized data sample using a stratified randomized sampling method [13]. This method reduces measurement variability and estimation errors, making it more effective than a simple random sampling [14]. The stratified sampling method generates credible data that effectively represent the entire population, improving the quality and validity of the research [13]. For this study, data from randomly sampled 20% of the total population that underwent PCI using DESs from 2002 to 2018 were provided by the Health Insurance Review and Assessment Service [15,16].

**Figure 1:**
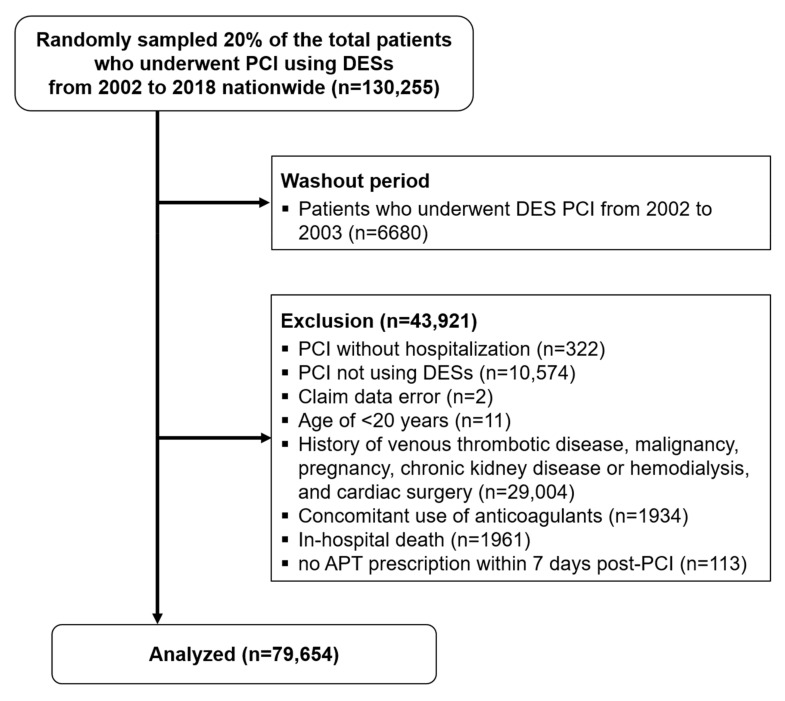
CONSORT flow diagram of the analyzed patients. DES, drug-eluting stent; PCI, percutaneous coronary intervention; APT, antiplatelet treatment.

A washout period of 2 years (2002–2004) was set to ensure greater internal validity. Among 123,575 patients, those with an identified history of venous thrombotic disease, malignancy, pregnancy, chronic kidney disease or hemodialysis, and cardiac surgery during the washout period (n = 29,004); those who underwent non-DES PCI (n = 10,574) or PCI without hospitalization (n = 322); those who died during hospitalization (n = 1961); those aged under 20 years (n = 11); those who were identified to be receiving no APT within 7 days after PCI (n = 113) or concomitant anticoagulants (n = 1934); and those with claim data error (n = 2) were excluded (Figure 1).

The index PCI date was the date of the first PCI from 1 January 2004 to 31 December 2018. Baseline clinical characteristics, medical history prior to PCI, and clinical diagnosis at the time of the index PCI [acute myocardial infarction (AMI) vs. non-AMI] were determined by respective International Classification of Diseases-10 codes [15]. We analyzed prescription claim data of five key antiplatelet agents, including aspirin, clopidogrel, ticagrelor, prasugrel, and cilostazol, collected at week and 1, 3, 6, 12, 18, and 24 months post-PCI and yearly, thereafter. The patients were considered censored at the time of the last available data, at the date of death, or when no APT claim was issued for >30 days during the first 6 months post-PCI or for >90 days thereafter.

### 2.2. APT Patterns and 15-Year Temporal Trends

**Figure 2:**
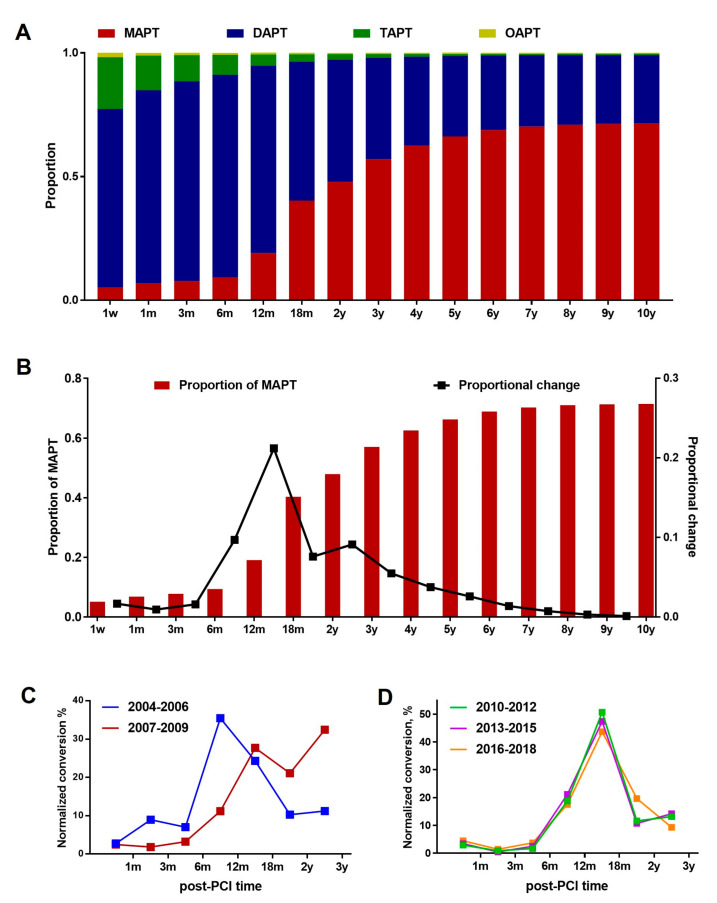
(**A**) Stacked bar graphs showing the relative proportion of the four different APT regimens (MAPT vs. DAPT vs. TAPT vs. OAPT) at each post-PCI time point. (**B**) Bar graph: MAPT prescription trend. Line graph: proportional increase in MAPT (switching to MAPT, %). (**C**,**D**) Time-course changes in the APT switching trends as assessed longitudinally at 3-year intervals.

The APT type was determined on the basis of the number and combination of prescribed antiplatelet agents. The patients were considered to receive MAPT when they were prescribed a single antiplatelet agent (aspirin or any P2Y12i); DAPT when aspirin plus any single P2Y12i were prescribed; triple antiplatelet treatment (TAPT), when three antiplatelet agents, including aspirin, clopidogrel, and cilostazol were prescribed; and other antiplatelet treatment (OAPT), if any two agents other than those described above were used. DAPT-to-MAPT conversion was assessed by calculating the proportional increases in MAPT use between two consecutive time points. To investigate the temporal changes in the APT patterns over 15 years, we subdivided and analyzed the entire data from 2004 to 2018 at 3-year intervals as follows: (1) 2004–2006, (2) 2007–2009, (3) 2010–2012, (4) 2013–2015, and (5) 2016–2018. The initial APT was determined on the basis of the antiplatelet agents that were prescribed at 3 months post-PCI. The long-term APT was defined based on the prescription data at 3 years post-PCI, given that a shift to monotherapy frequently occurred as late as between 2 and 3 years post-PCI (Figure 2B). The cases that were censored before the respective time points (e.g., due to death, right-censoring, or lack of claim data) were excluded from the analysis. The APT trends were analyzed according to the patient’s clinical characteristics, such as sex, clinical diagnosis (AMI vs. non-AMI), and age (<55, 55–64, 65–74, and >75 years).

### 2.3. Statistical Methods

Continuous variables are expressed as means ± standard deviations, and dichotomous variables as counts and percentages. The study population was categorized into the four APT types at each time point described above, and the data are presented as proportions. The relative risk test was performed to evaluate the effect of the clinical characteristics on the selection of the APT strategy. The outcomes were presented as relative risks and 95% confidence intervals. All statistical analyses were performed using SAS Enterprise Guide 7.1 (SAS Institute, Drive Cary, NC, USA). The statistical significance level was set at *p*-values of <0.05.

## 3. Results

### 3.1. Study Population

The data from a total of 79,654 patients were finally analyzed (Figure 1). The clinical characteristics of the study population are shown in Table 1. The mean patient age was 63.1 years, and the majority were men (n = 54,566, 68.5%). At the time of the index PCI, 38.7% of the patients presented with AMI (n = 30,823), and the mean number of implanted DES per patient was 1.27 ± 0.57. Among the 79,654 patients, 13,972 (17.5%) remained observable until 10 years post-PCI. The detailed statistics and information of the censored patients are presented in Figure S1 and Table S1.

### 3.2. Overall APT Prescription Patterns: MAPT vs. DAPT vs. TAPT

DAPT was the most preferred initial APT strategy, accounting for 80.7% of all APTs at 3 months post-PCI. DAPT proportion was 78.0% at 1-month post-PCI, peaked at 81.7% at 6 months, and decreased to 75.7% at 12 months, 56.1% at 18 months, 49.3% at 2 years, 41.0% at 3 years, and 32.4% at 5 years post-PCI. It remained grossly similar from 6 to 10 years, indicating that a substantial number of DES recipients (27.7% at 10 years post-PCI) received DAPT for a long time. Among them, the proportion of those who underwent repeated PCI was less than 5% (Figure S2). The proportion of TAPT as the initial APT was found to be rather high, being 21.0% at 1 week and 10.5% at 3 months post-PCI. However, it declined markedly thereafter: 8.0% at 6 months, 4.5% at 12 months, and below 1% at 5 years post-PCI. MAPT was the least preferred initial APT strategy, accounting for 6.8% at 1 month and 7.8% at 3 months post-PCI. The proportion reached over 55% from 3 years post-PCI and increased gradually up to 71.4% at 10 years post-PCI (Figure 2A).

### 3.3. DAPT-to-MAPT Conversion

In general, DAPT-to-MAPT conversion began between 6 and 12 months post-PCI (9.7%), and the largest increment in the proportion of MAPT occurred between 12 and 18 months (21.2%), reaching 40.3% at 18 months and 47.9% at 2 years post-PCI (Figure 2B, line graph). The conversion trends did not differ significantly between AMI and non-AMI individuals (Figure S3). In the longitudinal analysis at 3-year intervals, a remarkable difference in the conversion trends was noted between the mid-and-late 2000s (Figure 2C). Unlike the years 2004–2006, wherein DAPT-to-MAPT conversion occurred robustly between 6 and 12 months post-PCI, those of the year 2007–2009 began later and peaked as late as 3 years post-PCI (Figure 2C, red line). The conversion trends were grossly similar during the 2010s, occurring most robustly at 12–18 months post-PCI (Figure 2D).

### 3.4. Preferred Antiplatelet Agents for MAPT and Preferred Combination for DAPT

**Figure 3:**
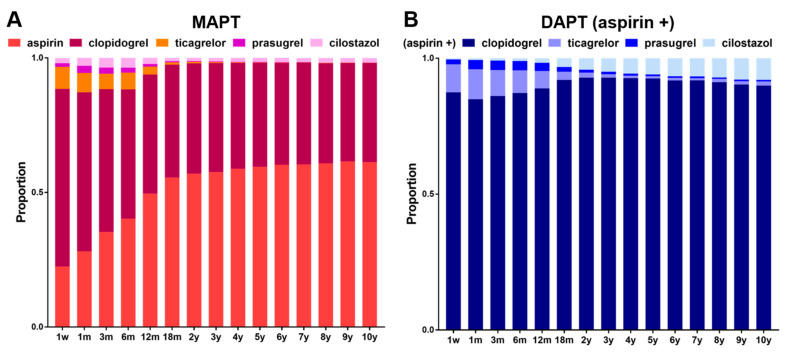
(**A**) Time-course proportion of antiplatelet agent used in the patients receiving mono-antiplatelet treatment (MAPT) and (**B**) drug combination in those receiving dual-antiplatelet treatment (DAPT). Detailed statistics are presented in Table S2.

Among the patients who received MAPT post-discharge, clopidogrel was the most frequently prescribed drug (66.1% at 1 week and 59.2% at 1 month), followed by aspirin (22.4% at 1 week and 28.1% at 1 month) and ticagrelor (8.2% at 1 week and 7.1% at 1 month) (Figure 3A). Prasugrel (1.3% at 1 week and 2.7% at 1 month) and cilostazol (1.9% at 1 week and 2.9% at 1 month) were rarely prescribed as monotherapy. From 1-year post-PCI, aspirin became the most frequently prescribed MAPT agent, accounting for 57.6% at 3 years. The proportion of aspirin use increased slightly but gradually thereafter, reaching 61.3% at 10 years. The second most common agent used for long-term MAPT was clopidogrel, accounting for 40.5% of the total prescriptions at 3 years and 36.9% at 10 years post-PCI (Figure 3A; detailed statistics are presented in Table S2).

Aspirin plus clopidogrel was the most preferred combination for DAPT (87.4% at 1 week and 88.9% at 1 year post-PCI) (Figure 3B). The preference for this combination persisted, accounting for 92.9% at 3 years and 89.9% at 10 years post-PCI. The second most frequently prescribed combination was aspirin plus ticagrelor (10.3% at 1 week and 6.4% at 1 year); however, the proportion of which started to decrease as early as 3 months and remained below 2% from 2 years post-PCI, reflecting trends of selecting ticagrelor-based DAPT for short-term use during the early post-PCI phase. Aspirin plus cilostazol was the least preferred combination as the initial DAPT (0.4% at 3 months post-PCI) but became the second most frequently prescribed regimen from 2 years post-PCI, with the proportion reaching 8.0% at 10 years (Figure 3B and Table S2). In general, aspirin plus prasugrel was the least preferred combination in Korea (Figure 3B; detailed statistics are presented in Table S2).

### 3.5. Changes in the APT Strategy during the Past 15 Years

**Figure 4:**
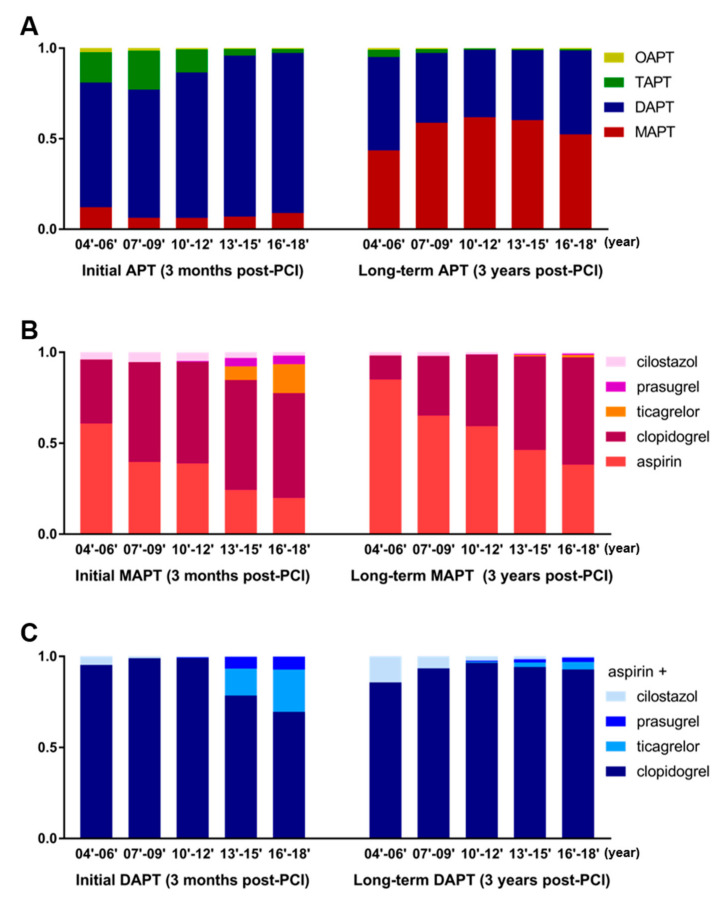
Longitudinal changes in APT selection as assessed at 3-year intervals between 2004 and 2018. (**A**) 15-year longitudinal trends in selecting MAPT, DAPT, and TAPT. (**B**,**C**) Time-course changes in the principal antiplatelet drugs that constitute the MAPT and DAPT regimens.

Given that the market release of newer P2Y12is began much later than that of clopidogrel (e.g., prasugrel in early 2011 and ticagrelor in early 2013 in Korea), we compared the prescription patterns between earlier and recent years (Figure 4). In the mid-to-late 2000s, cilostazol-based TAPT was rather widely used as the initial APT (Figure 4A, left). However, it became largely substituted by DAPT in the mid-2010s (Figure 4A), along with a marked increase in newer P2Y12i use (Figure 4B, left). Additionally, cilostazol was decreasingly used as a part of long-term DAPT over time (Figure 4A, right). Utilization of long-term DAPT decreased from 2004–2006 to 2010–2012 but recently increased again since 2013–2015 (Figure 4A, right).

Unlike in the earlier years (2004–2006), in which aspirin constituted the largest proportion, clopidogrel became the most preferred initial MAPT agent from 2007–2009 (55.0%, Figure 4B, left). Aspirin use decreased further with the emergence of newer P2Y12is (ticagrelor and prasugrel, 15.8% in 2016–2018, Figure 4B). Of note, as for long-term MAPT, clopidogrel surpassed aspirin as the most frequently used agent from the year 2013–2015.

The longitudinal analysis showed that the emergence of newer P2Y12is certainly encroached on the conventional clopidogrel-based initial DAPT (69.6% of the total DAPT in 2016–2018; ticagrelor: 23.2% and prasugrel: 7.2% at 3 months post-PCI, Figure 4C). However, the proportion of newer P2Y12i use as a part of long-term APT remained small, reflecting the preference for older, less potent P2Y12i as a maintenance DAPT agent (Figure 4C). The entire data and detailed statistics from 2004 to 2018 at 3-year intervals are presented in Figure S4.

### 3.6. Drug Preference and APT Strategy According to the Patients’ Clinical Characteristics

**Figure 5:**
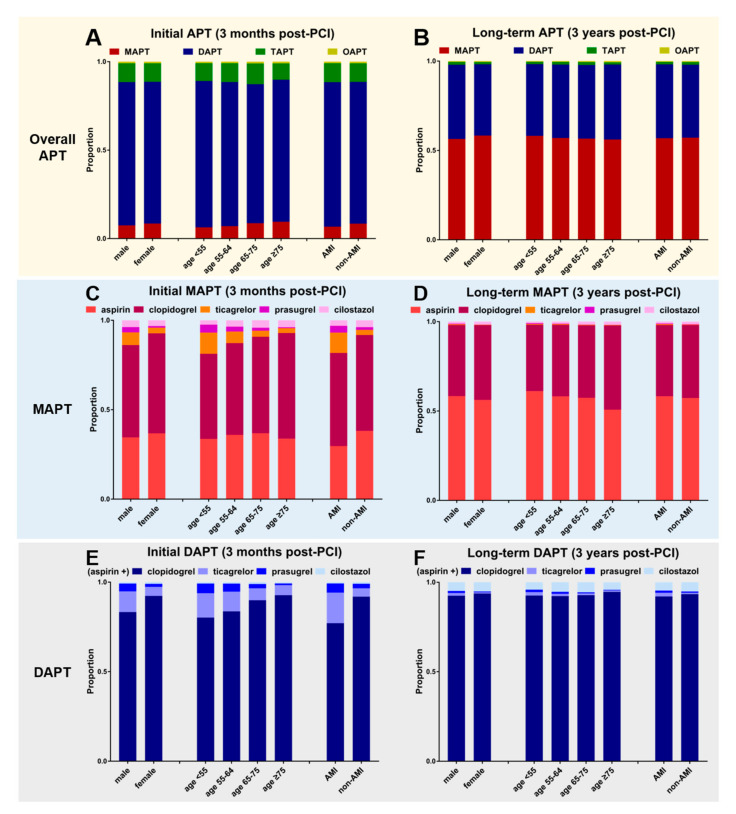
Selection of antiplatelet agents according to the patient’s clinical characteristics. (**A**,**B**) The proportion of MAPT, DAPT, and TAPT in each subgroup of DES recipients in the initial period (3 months post-PCI) and in the long-term (3 years post-PCI). (**C**,**D**) Antiplatelet agents among MAPT in each subgroup. (**E**,**F**) Antiplatelet combinations among DAPT in each subgroup.

The APT prescription patterns were evaluated according to sex, age, and clinical diagnosis (AMI vs. non-AMI) (Figure 5). The selection of the initial APT strategy (DAPT vs. MAPT vs. TAPT) appeared grossly similar (Figure 5A); however, the drugs that constituted each regimen differed significantly according to the patient’s clinical factors (Figure 5C,E). As for the initial APT, there were clear trends favoring potent P2Y12is for the patients with AMI, male sex, and younger age (Figure 5C,D). Meanwhile, no remarkable drug preference was noted in either long-term MAPT or long-term DAPT (Figure 5D,F), except for slight trends favoring aspirin in the younger population and clopidogrel in the older population as the long-term MAPT (Figure 5D). The entire data and detailed statistics according to clinical factors are presented in Figures S5–S7.

**Figure 6:**
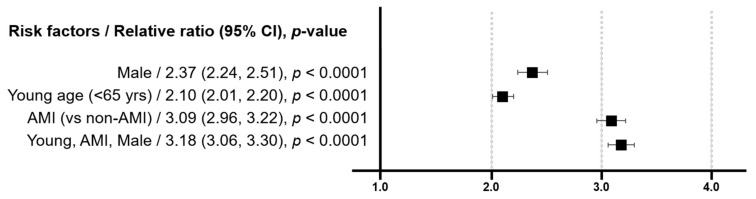
Forest plot showing the relative risk estimates and 95% CIs for the association between potent P2Y12 inhibitor use and the patient’s clinical characteristics. CI, confidence interval.

Male sex, younger age (<65 years), and AMI presentation were independently associated with an increased likelihood of receiving newer P2Y12i-based DAPT, with AMI presentation as the strongest determinant (Figure 6). In the multivariate analysis, the young male patients presenting with AMI were 3.18 times more likely to receive potent P2Y12i treatment than their counterparts (Figure 6).

## 4. Discussion

To the best of our knowledge, this study is one of the largest and longest studies to describe real-world APT in patients who underwent coronary DES implantation. Our systematic longitudinal analysis, including the key antiplatelet agents commonly used in the contemporary PCI era, allowed comprehensive interrogation of initial and long-term APTs, drug compositions, DAPT-to-MAPT conversion, and temporal changes in APT over a 15-year period. The principal findings are as follows: (1) A substantial number of patients were receiving prolonged DAPT for as long as over 10 years. (2) Clopidogrel surpassed aspirin as the most widely used agent in Korea. (3) A marked delay in DAPT-to-MAPT conversion was noted in the mid- to late 2000s, while conversion was grossly similar during the 2010s. (4) The emergence of newer P2Y12is certainly affected the APT strategy in recent years; however, their use was limited to a selective subset and peaked during the early post-PCI phase.

The proportion of prolonged DAPT herein (49.3% at 2 years post-PCI) is similar to those reported in the United States and Europe (43–57% at 2 years post-PCI); and decreased gradually with time [17,18]. However, the decline plateaued, and eventually, more than one-quarter of the patients (27.7%) remained on DAPT for over 10 years. Furthermore, a trend toward a re-increase in prolonged DAPT use was noted. The DAPT Study published in 2014 was a seminal trial that demonstrated an ischemic benefit of DAPT beyond 12 months, albeit at the expense of greater bleeding, compared to aspirin monotherapy [19]. This study provided a rationale to justify prolonged DAPT use in those with greater ischemic risks. However, with several subsequent RCTs showing conflicting results [20,21,22], meta-analyses provided inconclusive evidence on the effect of prolonged DAPT on all-cause mortality, with some study findings leaning toward rather an increased risk [23,24,25]. In contemporary PCI using ultrathin-strut DESs, intracoronary imaging, and high-dose statin administration, the ischemic benefit of prolonged DAPT might be further mitigated [5]. In this context, a recent study evaluated the applicability of the DAPT trial to contemporary practice and showed that extended DAPT was no longer effective in reducing ischemic events and still yielded an increased bleeding risk [26]. It is increasingly recognized that both bleeding and recurrent coronary ischemia are independent determinants of mortality after PCI, and the risk of major bleeding is comparable to or even greater than that of the ischemia [27,28]. A strategy of P2Y12i monotherapy after 1–3 months of DAPT has been recently proposed as an alternative to standard DAPT in patients undergoing complex PCI [29]. In this study based on a pooled analysis of 5 RCTs, P2Y12i monotherapy followed by a short DAPT course was associated with similar risks of ischemic events, regardless of PCI complexity, P2Y12i types, and clinical presentation; but with significantly lower bleeding risks compared to standard DAPT [29]. Although the current guidelines recommend a patient-tailored approach based on the combined ischemic and bleeding risks [7], it is often difficult to apply such a principle in routine practice. Our findings, coupled with recent evidence, highlight the need to raise physicians’ awareness of the potential hazard of prolonged DAPT and for an easily accessible automated decision support tool to guide clinicians to effectively screen those who might benefit from prolonged DAPT [30,31].

Aspirin has been recommended as the first-line drug for maintenance monotherapy after DES PCI. However, in our study, we found that clopidogrel had long been widely utilized in Korea and had recently surpassed aspirin as the most preferred agent for both initial and long-term MAPT. The HOST-EXAM trial, an RCT conducted in Korea, firstly demonstrated that long-term clopidogrel-based MAPT, as compared with aspirin monotherapy, significantly reduced the risk of the composite of all-cause death, myocardial infarction, stroke, readmission owing to coronary events, and bleeding events [8]. The accumulated clinical experience based on the large patient pool treated with long-term clopidogrel in Korea was certainly a motivator for initiating this trial. Although the ischemic benefit of clopidogrel over aspirin has been suggested, it is still under debate. Given that the HOST-EXAM trial studied relatively low-risk patients who tolerated 6–18 months of DAPT without any ischemic and major bleeding complications [8], further studies are warranted to extrapolate the findings into unselected patients with heterogeneous risk profiles.

There has been a lack of data reporting on the real-world APT trends with respect to the timing of switching to MAPT. First-generation DESs significantly reduced in-stent restenosis and repeat revascularization rates compared with bare metal stents [32]. However, the catastrophic occurrence of late stent thrombosis, especially after DAPT discontinuation [33], has raised concerns about not only the safety of first-generation DESs but also the adequacy of the recommended DAPT duration at that time: 3-month DAPT for sirolimus-eluting stents and 6-month DAPT for paclitaxel-eluting stents [34]. The revised 2007 guidelines recommended the extension of DAPT duration to at least 12 months or beyond. In our study, the concern was reflected in the noticeable delay in DAPT-to-MAPT conversion in the late 2000s. The observed delay was soon reversed, as newer stent designs using thinner, cobalt–chromium-based platforms and more bio-compatible polymers demonstrated enhanced safety [2,35]. However, although modern approaches, such as newer stent designs (e.g., ultrathin struts, biodegradable polymer, etc.), novel pharmacotherapeutics, or PCI optimization via fractional flow reserve or intracoronary imaging assessment, led to further improvement in clinical outcomes in recent years, DAPT-to-MAPT conversion did not appear to be altered during the past decade [2,5,35,36,37]. More updated data will address the concerns regarding whether recent efforts to shorten the DAPT duration are translated into the clinical practice [6].

Newer P2Y12is were developed to overcome the limitations of clopidogrel, such as a slow onset of action, drug irreversibility, and high interindividual variability [9,10]. In RCTs, both prasugrel and ticagrelor significantly reduced major adverse cardiovascular events compared with clopidogrel, although the agents were commonly associated with increased bleeding risks [9,10]. Thus, the guidelines were updated to recommend these drugs over clopidogrel in patients with acute coronary syndrome [6,7]. In the United States, ticagrelor was reported to surpass clopidogrel in 2017 as the most commonly used P2Y12i for patients with AMI [38]. A recent increase in ticagrelor use and a trend of ticagrelor-based DAPT replacing TAPT were noted; however, overall use of newer P2Y12is was still low in Korea (25.8% in 2013–2018). The patients treated with ticagrelor or prasugrel were younger, more likely to be men, and more likely to present with AMI than their counterparts, suggesting that physician-directed newer P2Y12i use remained limited to patients with low bleeding risks [39,40,41]. Global recommendations are largely based on RCTs in which East Asians were seldom enrolled. East Asians are known to have a greater susceptibility to bleeding and a lower likelihood of ischemic events than Westerners for any given level of platelet inhibition (“East Asian paradox”) [42,43,44]. Further, given that the proportion of platelet inhibition with newer P2Y12is is higher in East Asians than in Westerners [43], physicians in Korea might be reluctant to unconditionally apply the global guidelines [44]. Studies exploring the efficacy and safety of lower-dose ticagrelor or prasugrel will provide insights into optimal antiplatelet regimens for East Asians [45].

## 5. Study Limitations

This analysis based on an administrative claim dataset lacked detailed clinical information, including laboratory test findings, disease complexity, or behavioral risk factors, such as drinking and smoking. While this study was based on government claim data that universally covers the entire Korean population, the use of administrative data is unavoidably subject to various biases, such as selection bias, measurement bias, and reporting bias, which may potentially affect the validity of the results. Further, the possibility of some misentry or omission of diagnostic codes could not be discounted. Although we set a 2-year washout period and analyzed the patients who underwent repeated PCI separately, our analysis could not elucidate other reasons for prolonged DAPT use, such as ischemic stroke. Notably, cilostazol-based TAPT is not a guideline-recommended regimen and has been studied predominantly in Koreans. Lastly, the patients with a history of venous thrombotic disease, the most common thrombophilic condition, and those on anticoagulation therapy were screened to effectively exclude the participants with serious prothrombotic conditions. However, the study did not specifically identify patients with a wide variety of coagulation disorders or those with hemophilic conditions.

## 6. Conclusions

We spent over a decade in search of the optimal APT strategy following PCI using DESs. This study showed that the real-world APT prescription pattern is evolving, and gaps between guidelines and practice exist. The differences in the ischemic/bleeding threshold and varying responses to APT among ethnic groups might serve as hurdles for the generalized application of global guidelines. Further studies exploring the effect of the aforementioned APT strategies on the real-world outcomes of unselected patients are expected to provide insights into individualized APTs that can sophisticatedly balance the ischemic and bleeding risks.

## Figures and Tables

**Table 1:** Baseline Characteristics of the Study Population.

Variables	N	%
**Total patients**	79,654	
**Age, mean**	63.06 ± 11.53	
<55 years	19,315	24.2%
55–64 years	23,372	29.3%
65–74 years	23,094	29.0%
≥75 years	13,873	17.4%
**Sex**		
Male	54,566	68.5%
Female	25,088	31.5%
**Hospital type**		
Tertiary hospital	39,794	50.0%
Secondary general hospital	39,768	49.9%
Primary clinic	92	0.1%
**Clinical diagnosis at the index PCI**		
Acute myocardial infarction	30,823	38.7%
Non-acute myocardial infarction	48,831	61.3%
**Comorbidities**		
Diabetes mellitus	26,140	32.8%
Hypertension	43,489	54.6%
Dyslipidemia	31,833	40.0%
Peripheral vascular disease	12,109	15.2%
Stroke (ischemic or hemorrhagic)	5590	7.0%

PCI, percutaneous coronary intervention.

## Data Availability

The data presented in this study are available at a reasonable request from the corresponding author. More data are contained within the Supplementary Materials.

## Supplementary Materials

The following supporting information can be downloaded at: https://www.mdpi.com/article/10.3390/jcm12072675/s1, Figure S1: Relative proportion of the patients receiving four different antiplatelet therapy and those of censored cases at each post-PCI time point; Table S1: Numbers and percentages of the patients receiving four different antiplatelet therapy; Figure S2: Relative proportion of the different APT regimens at each post-PCI time point; Figure S3: The trends in DAPT-to-monotherapy conversion between patients with acute myocardial infarction and those without infarction; Table S2: Detailed statistics of antiplatelet therapies over time; Figure S4: Trends in antiplatelet treatment from 2004 to 2018 at 3-year intervals; Figure S5: Trends in antiplatelet treatment in each sex group; Figure S6: Trends in antiplatelet treatment in each age group; Figure S7: Trends in antiplatelet treatment in AMI and non-AMI patients.
