# Supplementary material for: Fifteen-Year Nationwide Trend in Antiplatelet Treatment among Drug-Eluting Stent Recipients in Korea: Many Patients Receive Very Prolonged Dual-Antiplatelet Treatment, and Newer Drugs Are Replacing the Older Ones
Source: J Clin Med. 2023 Apr 3;12(7):2675. doi: 10.3390/jcm12072675 (PMC10095404; doi:10.3390/jcm12072675)
Supplement: Supplementary file 1 [file jcm-12-02675-s001.zip › jcm-2286939-supplementary.pdf]

SUPPLEMENTAL MATERIALS

Fifteen-Year Nationwide Trend in Antiplatelet Treatment among Drug-Eluting Stent Recipients in Korea: Many Patients Receive Very Prolonged Dual-Antiplatelet Treatment, and Newer Drugs Are Replacing the Older Ones

Sunwon Kim, Jong-Seok Lee, Jungkuk Lee, Yong-Hyun Kim, Jin-Seok Kim, Sang-Yup Lim, Seong Hwan Kim, Jeong-Cheon Ahn and Woo-Hyuk Song

Cardiovascular Center, Korea University Ansan Hospital, Ansan-si, South Korea

Supplementary Results

Study population: A total of 79,654 patients' data was finally analyzed. 6,658 patients were censored as death; 20,975 were due to absence of antiplatelet prescription; and 41,545 patients were right-censored (type I censoring) during 10 years of observation period. 13,972 patients remained observable until post-PCI tenth year. Mortality rate was 1825.8 per 100,000 person-years (**Figure S1**).

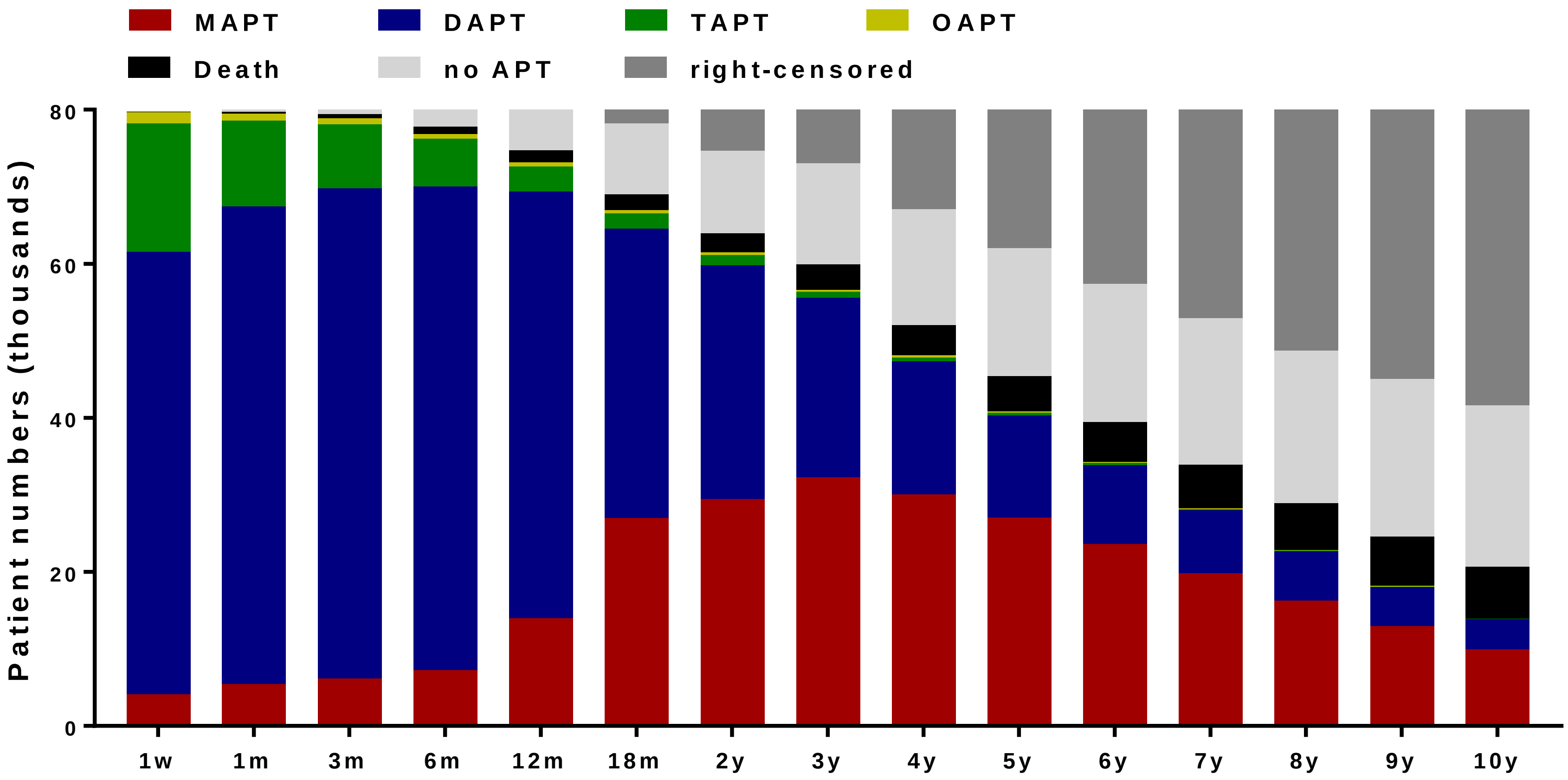

Figure S1. Stacked bar graphs showing the relative proportion of the patients receiving four different antiplatelet therapy and those of censored cases at each post-PCI time point. MAPT = mono-antiplatelet treatment; DAPT = dual-antiplatelet treatment; TAPT = triple-antiplatelet treatment; OAPT = other antiplatelet treatment. Detailed statistics are presented in **Table S1**.

Date	Total	Death	No APT	Right-censored	MAPT		DAPT (aspirin +)		TAPT (aspirin + clopidogrel +)		OAPT	
	n	n	n	n	n	%	n	%	n	%	n	%
1 week	79654	90	83	2	4112	5.2%	57417	72.1%	16713	21.0%	1412	1.8%
1 month	79479	170	424	10	5439	6.8%	62015	78.0%	11122	14.0%	903	1.1%
3 months	78875	314	1326	386	6143	7.8%	63681	80.7%	8301	10.5%	750	1.0%
6 months	76849	340	2492	839	7213	9.4%	62806	81.7%	6184	8.0%	646	0.8%
12 months	73178	647	2962	2613	13965	19.1%	55401	75.7%	3300	4.5%	512	0.7%
18 months	66956	488	1922	3064	26980	40.3%	37561	56.1%	1993	3.0%	422	0.6%
2 years	61482	438	1509	2871	29451	47.9%	30334	49.3%	1348	2.2%	349	0.6%
3 years	56664	805	2360	5430	32310	57.0%	23241	41.0%	815	1.4%	298	0.5%
4 years	48069	682	1957	4596	30048	62.5%	17282	36.0%	510	1.1%	229	0.5%
5 years	40834	624	1554	4385	27059	66.3%	13238	32.4%	333	0.8%	204	0.5%
6 years	34271	565	1377	4087	23600	68.9%	10274	30.0%	235	0.7%	162	0.5%
7 years	28242	483	1047	3854	19838	70.2%	8132	28.8%	146	0.5%	126	0.4%
8 years	22858	411	804	3454	16226	71.0%	6435	28.2%	110	0.5%	87	0.4%
9 years	18189	345	649	3223	12968	71.3%	5049	27.8%	87	0.5%	85	0.5%
10 years	13972	256	509	2731	9978	71.4%	3873	27.7%	66	0.5%	55	0.4%

Table S1. Numbers and percentages of the patients receiving four different antiplatelet therapy and those of censored cases at each post-PCI time point. MAPT = mono-antiplatelet treatment; DAPT = dual-antiplatelet treatment; TAPT = triple-antiplatelet treatment; OAPT = other antiplatelet treatment.

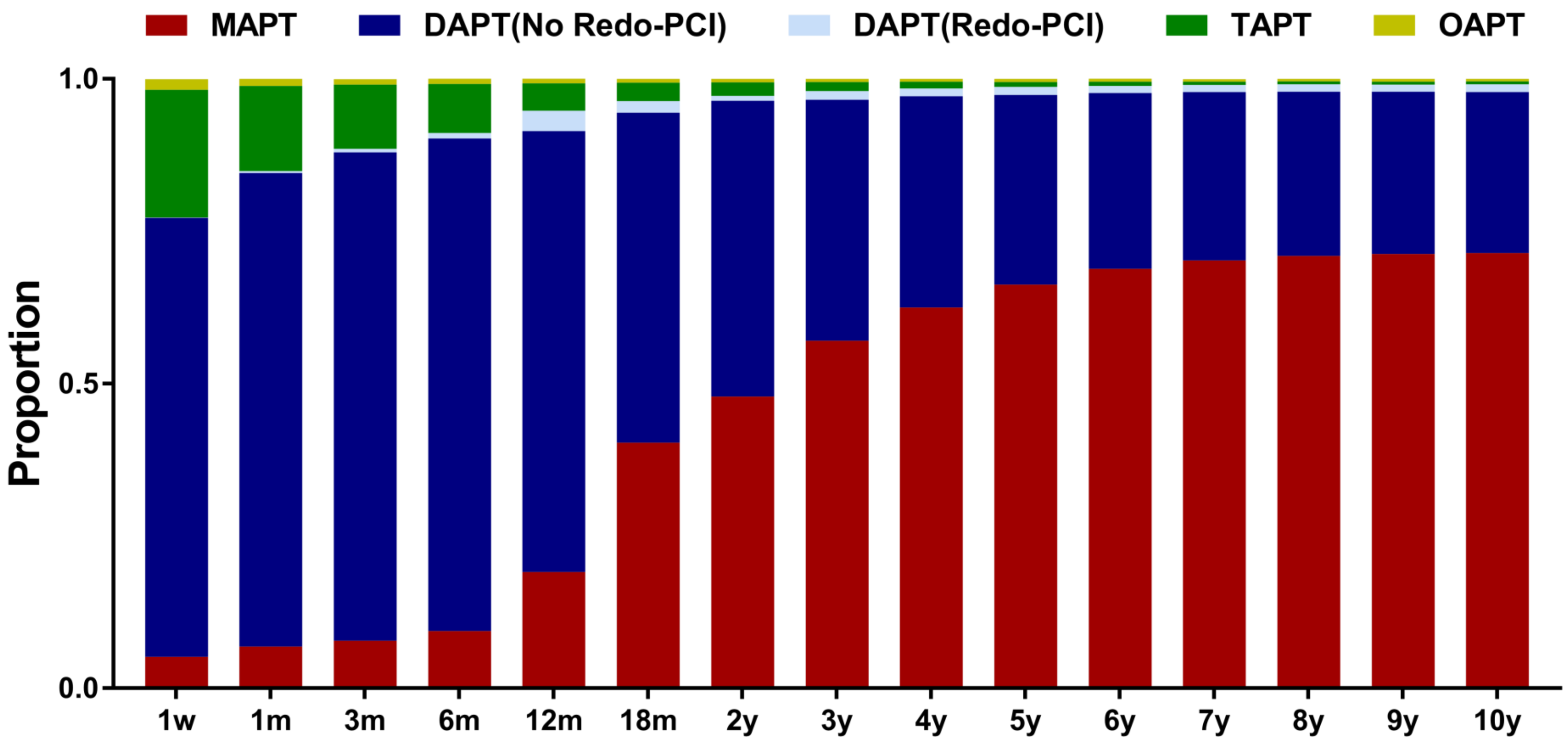

Figure S2. Stacked bar graph showing the relative proportion of the different APT regimens (MAPT vs. DAPT vs. TAPT vs. OAPT) at each post-PCI time-point. Among them, the proportion of those who underwent repeated PCI (redo-PCI) was less than 5%.

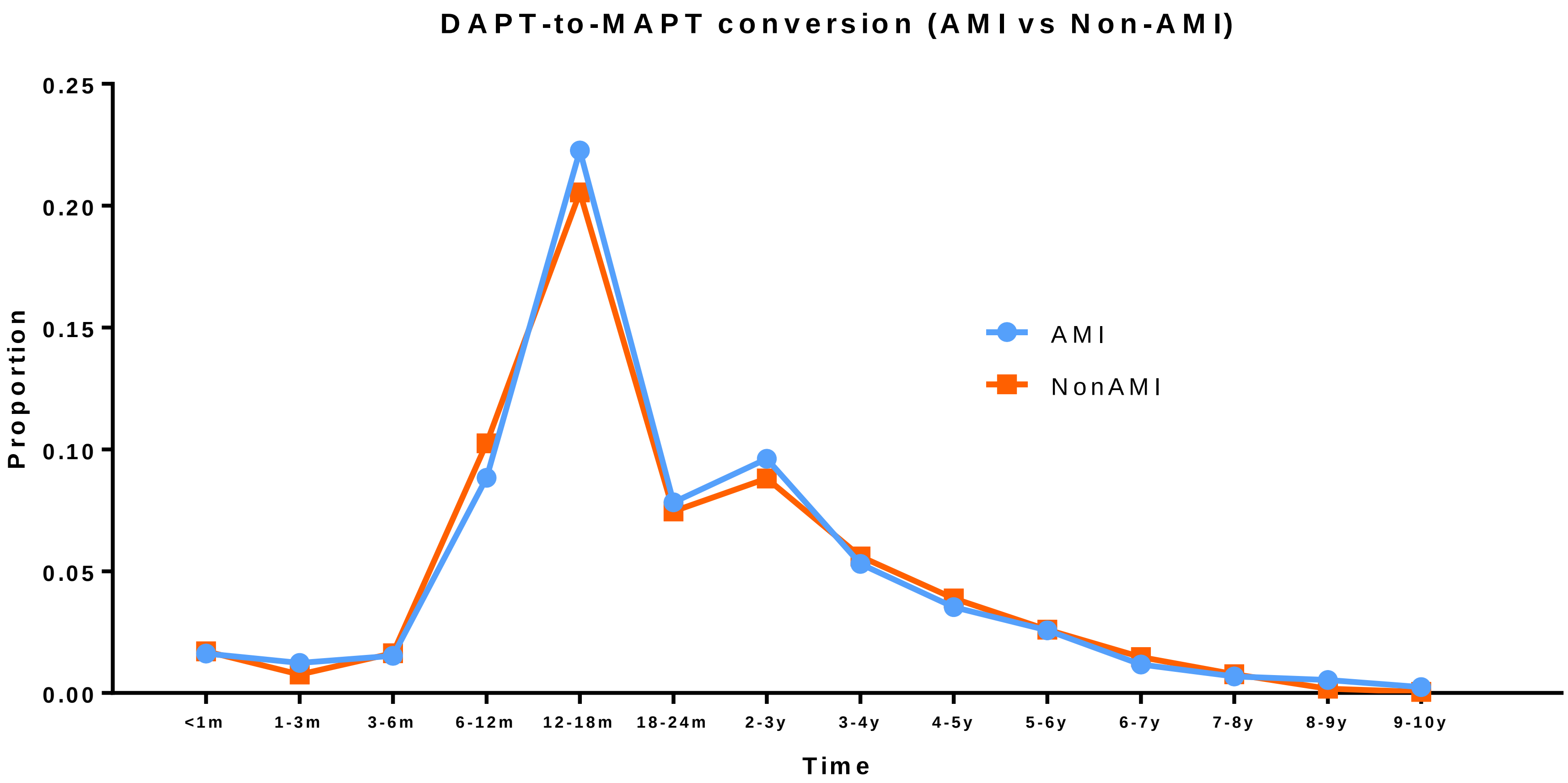

Figure S3. The trends in DAPT-to-monotherapy conversion between patients with acute myocardial infarction and those without infarction.

Date	Total	MAPT	DAPT	TAPT	OAPT	Death	No APT	Right-censored	MAPT					DAPT (aspirin +)			
	N					n	n	n	asp	clop	tica	pra	cilo	clop	tica	pra	cilo
1w	79654	5.2%	72.1%	21.0%	1.8%	90	83	2	22.4%	66.1%	8.2%	1.3%	1.9%	87.4%	10.3%	1.8%	0.4%
1m	79479	6.8%	78.0%	14.0%	1.1%	170	424	10	28.1%	59.2%	7.1%	2.7%	2.9%	84.9%	11.0%	3.4%	0.7%
3m	78875	7.8%	80.7%	10.5%	1.0%	314	1326	386	35.3%	53.1%	5.7%	2.3%	3.6%	86.1%	9.5%	3.5%	0.9%
6m	76849	9.4%	81.7%	8.0%	0.8%	340	2492	839	40.3%	48.0%	6.2%	1.9%	3.6%	87.2%	8.3%	3.5%	1.0%
12m	73178	19.1%	75.7%	4.5%	0.7%	647	2962	2613	49.6%	44.3%	2.9%	1.0%	2.2%	88.9%	6.4%	3.0%	1.7%
18m	66956	40.3%	56.1%	3.0%	0.6%	488	1922	3064	55.6%	41.9%	0.9%	0.4%	1.2%	92.0%	3.0%	1.7%	3.2%
2y	61482	47.9%	49.3%	2.2%	0.6%	438	1509	2871	57.0%	41.0%	0.5%	0.3%	1.2%	92.8%	1.8%	1.1%	4.2%
3y	56664	57.0%	41.0%	1.4%	0.5%	805	2360	5430	57.6%	40.5%	0.3%	0.3%	1.3%	92.9%	1.3%	0.9%	4.9%
4y	48069	62.5%	36.0%	1.1%	0.5%	682	1957	4596	58.8%	39.4%	0.2%	0.2%	1.4%	92.7%	0.9%	0.8%	5.7%
5y	40834	66.3%	32.4%	0.8%	0.5%	624	1554	4385	59.5%	38.7%	0.1%	0.2%	1.5%	92.6%	0.8%	0.7%	5.9%
6y	34271	68.9%	30.0%	0.7%	0.5%	565	1377	4087	60.3%	37.9%	0.1%	0.1%	1.6%	91.8%	0.9%	0.6%	6.6%
7y	28242	70.2%	28.8%	0.5%	0.4%	483	1047	3854	60.4%	37.9%	0.1%	0.1%	1.5%	91.9%	0.8%	0.6%	6.7%
8y	22858	71.0%	28.2%	0.5%	0.4%	411	804	3454	60.8%	37.2%	0.1%	0.1%	1.8%	91.2%	1.2%	0.5%	7.1%
9y	18189	71.3%	27.8%	0.5%	0.5%	345	649	3223	61.5%	36.6%	0.1%	0.0%	1.7%	90.3%	1.2%	0.6%	7.8%
10y	13972	71.4%	27.7%	0.5%	0.4%	256	509	2731	61.3%	36.9%	0.1%	0.0%	1.7%	89.9%	1.5%	0.6%	8.0%

Table S2. Detailed statistics of antiplatelet therapies over time, shown as the numbers of patients and proportions of each antiplatelet regimen among MAPT and DAPT (n=number of patients, %=proportion of each antiplatelet regimen among the type, asp=aspirin, clop=clopidogrel, tica=ticagrelor, pra=prasugrel, cilo=cilostazol)

A. Trends in antiplatelet treatment in year 2004-2006.

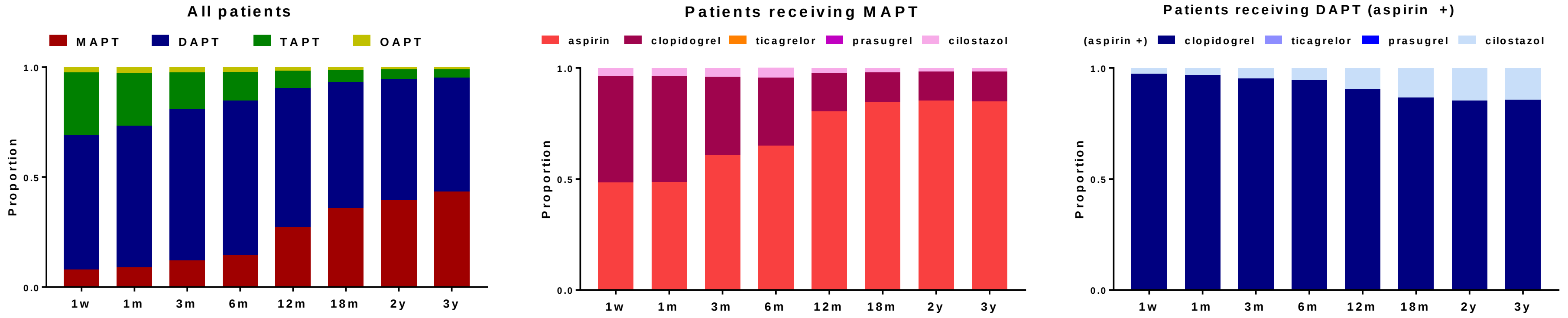

Date	Total	MAPT	DAPT	TAPT	OAPT	Death	No APT	Right-censored	MAPT					DAPT (aspirin +)			
	n					n	n	n	asp	clop	tica	pra	cilo	clop	tica	pra	cilo
1 week	10124	8.0%	61.4%	28.2%	2.5%	19	9	0	48.4%	47.8%	0.0%	0.0%	3.7%	97.4%	0.0%	0.0%	2.6%
1 month	10096	8.9%	64.4%	24.2%	2.5%	21	62	0	48.6%	47.7%	0.0%	0.0%	3.8%	96.8%	0.0%	0.0%	3.2%
3 months	10013	12.1%	68.9%	16.7%	2.4%	44	203	0	60.8%	35.3%	0.0%	0.0%	4.0%	95.3%	0.0%	0.0%	4.7%
6 months	9766	14.6%	70.3%	13.0%	2.1%	38	489	0	65.0%	30.7%	0.0%	0.0%	4.3%	94.4%	0.0%	0.0%	5.6%
12 months	9239	27.2%	63.4%	7.8%	1.5%	93	511	0	80.4%	17.2%	0.0%	0.0%	2.4%	90.6%	0.0%	0.0%	9.4%
18 months	8635	35.8%	57.5%	5.5%	1.1%	68	245	0	84.5%	13.5%	0.0%	0.0%	2.0%	86.7%	0.0%	0.0%	13.3%
2 years	8322	39.5%	55.1%	4.4%	1.0%	70	201	0	85.2%	13.2%	0.0%	0.0%	1.6%	85.2%	0.0%	0.0%	14.8%
3 years	8051	43.5%	51.6%	3.9%	0.9%	121	346	0	84.9%	13.3%	0.0%	0.0%	1.7%	85.7%	0.0%	0.0%	14.3%

B. Trends in antiplatelet treatment in year 2007-2009.

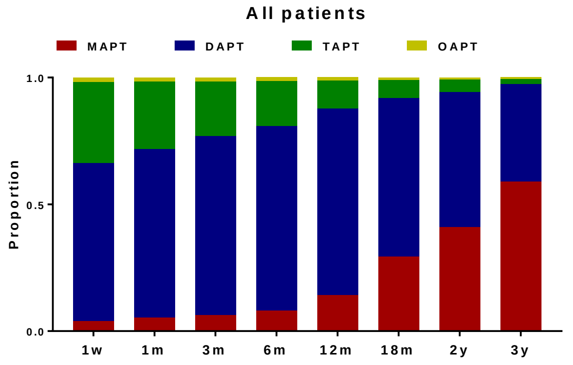

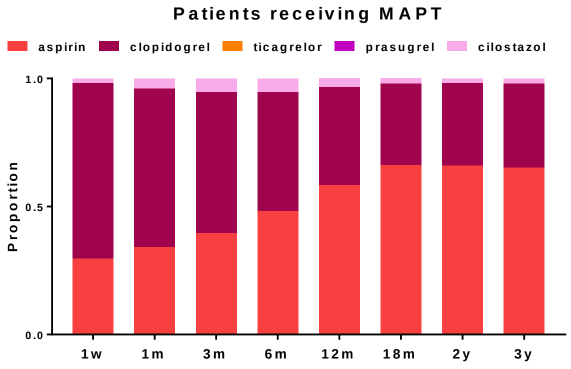

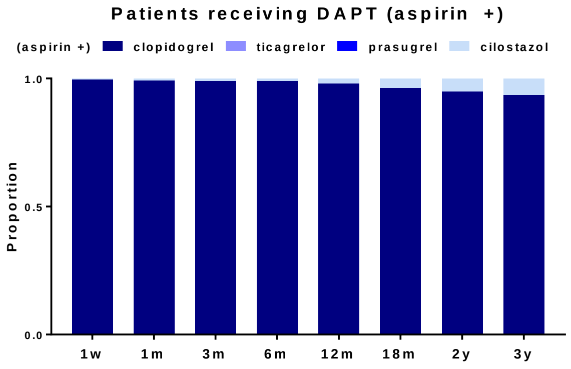

Date	Total	MAPT	DAPT	TAPT	OAPT
	n				
1 week	15813	3.9%	62.5%	32.0%	1.7%
1 month	15789	5.2%	66.6%	26.5%	1.7%
3 months	15701	6.3%	70.8%	21.5%	1.5%
6 months	15417	8.0%	72.8%	17.8%	1.3%
12 months	14723	14.2%	73.5%	11.2%	1.1%
18 months	13928	29.4%	62.3%	7.3%	1.0%
2 years	13438	41.0%	53.3%	4.9%	0.8%
3 years	12967	58.8%	38.5%	2.1%	0.6%

Death	No APT	Right-censored
n	n	n
19	5	0
32	56	0
61	223	0
73	621	0
141	654	0
104	386	0
104	367	0
234	608	0

MAPT					DAPT (aspirin +)			
asp	clop	tica	pra	cilo	clop	tica	pra	cilo
29.5%	68.7%	0.0%	0.0%	1.8%	99.5%	0.0%	0.0%	0.5%
34.1%	62.0%	0.0%	0.0%	4.0%	99.1%	0.0%	0.0%	0.9%
39.6%	55.0%	0.0%	0.0%	5.4%	99.0%	0.0%	0.0%	1.0%
48.2%	46.5%	0.0%	0.0%	5.3%	98.9%	0.0%	0.0%	1.1%
58.3%	38.2%	0.0%	0.0%	3.5%	98.0%	0.0%	0.0%	2.0%
66.1%	31.9%	0.0%	0.0%	2.1%	96.2%	0.0%	0.0%	3.8%
65.9%	32.4%	0.0%	0.0%	1.8%	94.8%	0.0%	0.0%	5.2%
65.1%	32.9%	0.0%	0.0%	2.0%	93.5%	0.0%	0.0%	6.5%

C. Trends in antiplatelet treatment in year 2010-2012.

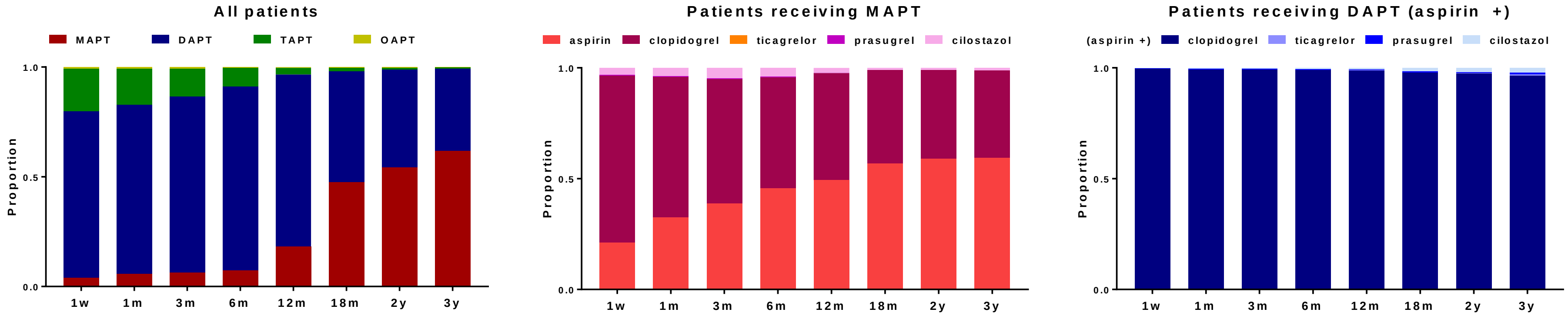

Date	Total	MAPT	DAPT	TAPT	OAPT
	n				
1 week	17472	4.0%	75.7%	19.5%	0.8%
1 month	17437	5.7%	77.1%	16.4%	0.8%
3 months	17323	6.3%	80.3%	12.8%	0.7%
6 months	16999	7.3%	83.9%	8.3%	0.5%
12 months	16309	18.2%	78.4%	3.0%	0.4%
18 months	15453	47.5%	50.6%	1.6%	0.3%
2 years	14848	54.2%	44.6%	0.9%	0.3%
3 years	14340	61.8%	37.3%	0.6%	0.2%

Date	Death	No APT	Right-censored
	n	n	n
1 week	27	8	0
1 month	42	72	0
3 months	77	247	0
6 months	105	585	0
12 months	161	695	0
18 months	121	484	0
2 years	109	399	0
3 years	216	625	0

Date	MAPT					DAPT (aspirin +)			
	asp	clop	tica	pra	cilo	clop	tica	pra	cilo
1 week	21.1%	75.4%	0.0%	0.4%	3.1%	99.5%	0.0%	0.3%	0.2%
1 month	32.4%	63.5%	0.0%	0.4%	3.7%	99.1%	0.0%	0.4%	0.5%
3 months	38.8%	55.9%	0.0%	0.5%	4.8%	99.1%	0.0%	0.4%	0.5%
6 months	45.6%	50.2%	0.0%	0.2%	4.0%	99.0%	0.0%	0.4%	0.5%
12 months	49.3%	48.3%	0.0%	0.1%	2.3%	98.8%	0.1%	0.4%	0.7%
18 months	56.9%	42.1%	0.0%	0.0%	1.0%	97.7%	0.2%	0.4%	1.7%
2 years	58.9%	40.0%	0.0%	0.0%	1.0%	97.4%	0.3%	0.4%	1.9%
3 years	59.3%	39.5%	0.1%	0.0%	1.2%	96.5%	0.6%	0.6%	2.3%

D. Trends in antiplatelet treatment in year 2013-2015.

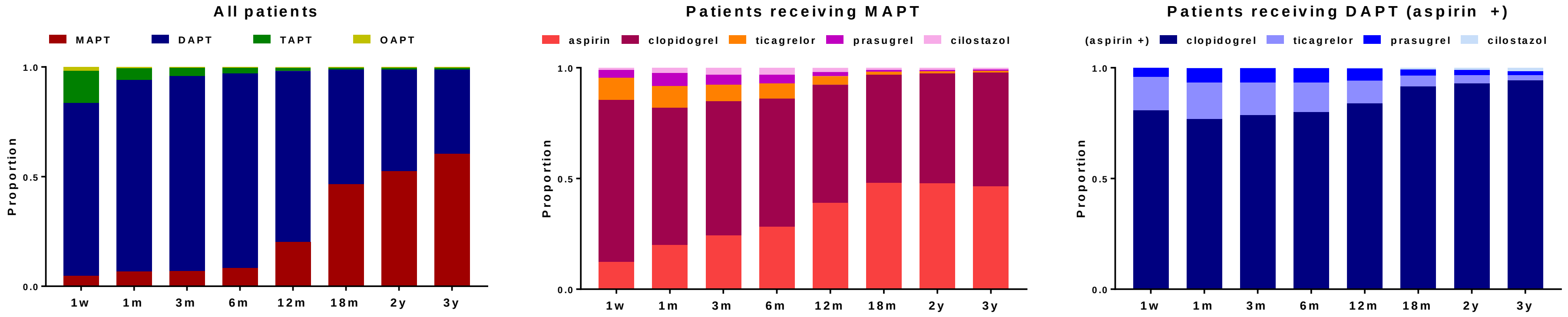

Date	Total	MAPT	DAPT	TAPT	OAPT	Death	No APT	Right-censored	MAPT					DAPT (aspirin +)			
	n					n	n	n	asp	clop	tica	pra	cilo	clop	tica	pra	cilo
1 week	17448	4.8%	78.9%	14.6%	1.8%	12	9	0	12.3%	73.2%	10.0%	3.6%	1.0%	80.8%	15.1%	4.1%	0.0%
1 month	17427	6.7%	87.5%	5.2%	0.7%	54	64	0	19.9%	62.1%	9.7%	5.9%	2.4%	76.8%	16.4%	6.6%	0.2%
3 months	17309	7.0%	88.9%	3.7%	0.5%	71	243	0	24.3%	60.5%	7.5%	4.6%	3.1%	78.6%	14.7%	6.5%	0.2%
6 months	16995	8.4%	88.6%	2.6%	0.4%	74	383	0	28.2%	57.9%	6.8%	4.0%	3.1%	79.9%	13.5%	6.4%	0.2%
12 months	16538	20.1%	78.1%	1.4%	0.4%	162	580	0	39.0%	53.4%	3.8%	1.9%	1.9%	83.9%	10.2%	5.5%	0.3%
18 months	15796	46.4%	52.3%	0.9%	0.4%	134	506	0	47.9%	48.9%	1.3%	0.8%	1.0%	91.6%	4.9%	2.8%	0.8%
2 years	15156	52.4%	46.5%	0.7%	0.4%	111	408	0	47.7%	49.7%	0.9%	0.6%	1.0%	92.9%	3.8%	2.2%	1.1%
3 years	14637	60.3%	38.6%	0.6%	0.5%	196	711	8	46.3%	51.5%	0.7%	0.6%	0.9%	94.2%	2.5%	1.8%	1.5%

E. Trends in antiplatelet treatment in year 2016-2018.

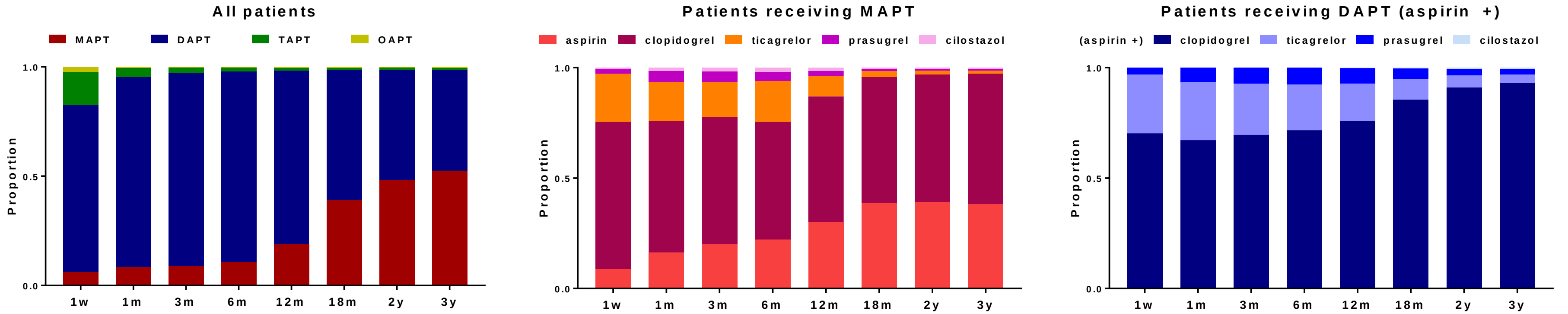

Date	Total	MAPT	DAPT	TAPT	OAPT	Death	No APT	Right-censored	MAPT					DAPT (aspirin +)			
	n					n	n	n	asp	clop	tica	pra	cilo	clop	tica	pra	cilo
1 week	18797	6.2%	76.2%	15.2%	2.4%	13	52	2	8.8%	66.6%	22.0%	1.9%	0.8%	70.1%	26.8%	3.1%	0.0%
1 month	18730	8.2%	87.1%	4.0%	0.7%	21	170	10	16.3%	59.4%	17.8%	4.8%	1.7%	67.0%	26.4%	6.5%	0.1%
3 months	18529	8.9%	88.4%	2.3%	0.4%	61	410	386	19.9%	57.8%	15.8%	4.8%	1.8%	69.6%	23.2%	7.2%	0.1%
6 months	17672	10.7%	87.2%	1.7%	0.4%	50	414	839	22.1%	53.3%	18.6%	3.9%	2.1%	71.6%	20.7%	7.5%	0.1%
12 months	16369	18.8%	79.5%	1.2%	0.5%	90	522	2613	30.1%	56.8%	9.2%	2.3%	1.6%	75.9%	16.9%	7.0%	0.2%
18 months	13144	39.0%	59.5%	0.9%	0.6%	61	301	3064	38.8%	57.0%	2.6%	1.0%	0.7%	85.5%	9.2%	4.9%	0.4%
2 years	9718	48.1%	50.6%	0.8%	0.5%	44	134	2871	39.1%	57.8%	1.6%	0.8%	0.6%	91.0%	5.4%	3.1%	0.5%
3 years	6669	52.4%	46.3%	0.7%	0.6%	38	70	5422	38.2%	59.1%	1.3%	0.7%	0.7%	92.8%	4.0%	2.5%	0.6%

Figure S4. Trends in antiplatelet treatment from 2004 to 2018 at 3-year intervals. Stacked bar graphs showing the relative proportion of the four different antiplatelet treatment in the patients who underwent DES implantation in year 2016-2018 (left panel). Proportion of antiplatelet agents prescribed for the patients who received mono-antiplatelet treatment (MAPT, middle panel) and those receiving dual-antiplatelet treatment (DAPT, right panel). asp = aspirin; clop = clopidogrel; tica = ticagrelor; pra = prasugrel; cilo = cilostazol

A. Trends in antiplatelet treatment in male patients.

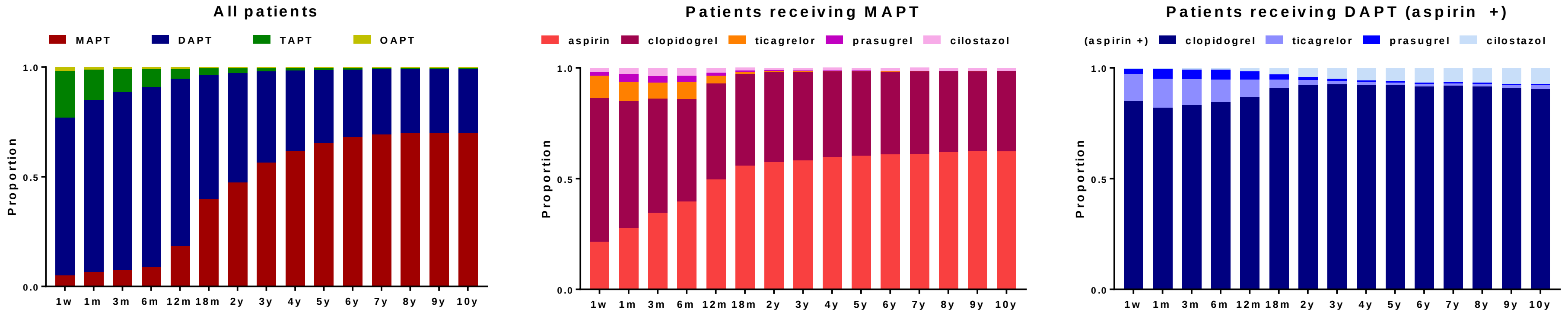

Date	Total	MAPT	DAPT	TAPT	OAPT
	n				
1 week	54566	5.0%	72.0%	21.1%	1.9%
1 month	54460	6.6%	78.4%	13.9%	1.1%
3 months	54038	7.5%	81.0%	10.6%	0.9%
6 months	52677	8.9%	82.1%	8.2%	0.8%
12 months	50181	18.3%	76.3%	4.7%	0.7%
18 months	45829	39.8%	56.5%	3.1%	0.6%
2 years	41901	47.4%	49.7%	2.3%	0.5%
3 years	38519	56.4%	41.6%	1.5%	0.5%
4 years	32500	61.7%	36.7%	1.1%	0.5%
5 years	27498	65.3%	33.3%	0.9%	0.5%
6 years	23017	68.1%	30.7%	0.7%	0.5%
7 years	18935	69.3%	29.7%	0.5%	0.5%
8 years	15288	69.8%	29.2%	0.5%	0.4%
9 years	12156	70.0%	28.9%	0.5%	0.5%
10 years	9364	70.1%	29.0%	0.5%	0.4%

Death	No APT	Right-censored
n	n	n
37	24	0
77	103	2
173	410	79
196	614	157
305	647	476
236	377	535
217	335	524
367	471	913
293	406	699
246	298	667
210	252	569
170	189	450
123	130	366
102	102	318
62	73	221

MAPT					DAPT (aspirin +)			
asp	clop	tica	pra	cilo	clop	tica	pra	cilo
21.5%	64.9%	10.0%	1.8%	1.9%	84.9%	12.4%	2.4%	0.4%
27.5%	57.5%	8.7%	3.6%	2.8%	81.9%	13.2%	4.3%	0.6%
34.5%	51.6%	7.1%	3.0%	3.8%	83.3%	11.6%	4.4%	0.8%
39.6%	46.4%	7.8%	2.6%	3.6%	84.5%	10.2%	4.3%	0.9%
49.6%	43.2%	3.7%	1.3%	2.1%	86.9%	7.8%	3.8%	1.6%
55.9%	41.3%	1.1%	0.5%	1.2%	91.1%	3.7%	2.2%	3.1%
57.5%	40.4%	0.6%	0.4%	1.1%	92.3%	2.2%	1.4%	4.1%
58.3%	39.8%	0.4%	0.3%	1.1%	92.5%	1.5%	1.1%	4.9%
59.8%	38.4%	0.3%	0.2%	1.3%	92.4%	1.1%	0.9%	5.7%
60.4%	38.0%	0.2%	0.2%	1.3%	92.2%	1.0%	0.8%	6.0%
61.0%	37.3%	0.1%	0.2%	1.4%	91.6%	1.1%	0.7%	6.6%
61.2%	37.3%	0.1%	0.1%	1.4%	92.0%	0.9%	0.7%	6.5%
61.9%	36.4%	0.1%	0.1%	1.4%	91.5%	1.3%	0.5%	6.7%
62.4%	36.1%	0.1%	0.0%	1.4%	90.8%	1.4%	0.7%	7.2%
62.4%	36.1%	0.1%	0.0%	1.4%	90.4%	1.8%	0.5%	7.3%

B. Trends in antiplatelet treatment in female patients.

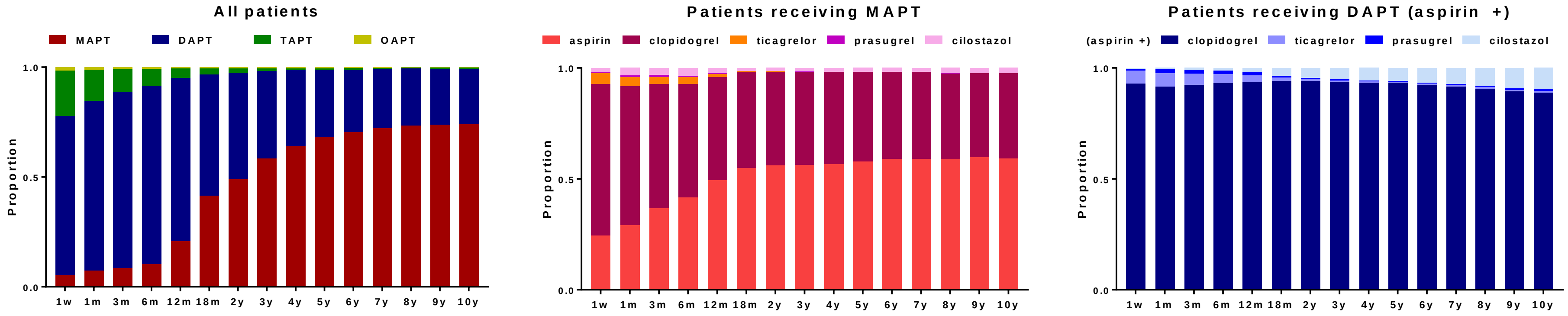

Date	Total	MAPT	DAPT	TAPT	OAPT	Death	No APT	Right-censored	MAPT					DAPT (aspirin +)			
	n					n	n	n	asp	clop	tica	pra	cilo	clop	tica	pra	cilo
1 week	25088	5.5%	72.3%	20.6%	1.6%	46	23	0	24.4%	68.4%	4.7%	0.4%	2.0%	93.0%	5.8%	0.6%	0.5%
1 month	25019	7.4%	77.3%	14.2%	1.2%	69	110	3	29.2%	62.5%	4.1%	1.0%	3.2%	91.5%	6.1%	1.6%	0.7%
3 months	24837	8.5%	80.2%	10.4%	1.0%	130	431	104	36.8%	55.9%	3.1%	0.9%	3.2%	92.3%	5.0%	1.7%	1.0%
6 months	24172	10.4%	81.0%	7.7%	0.9%	142	821	212	41.7%	51.0%	3.1%	0.6%	3.6%	93.1%	4.1%	1.6%	1.2%
12 months	22997	20.7%	74.4%	4.2%	0.7%	239	889	742	49.5%	46.3%	1.5%	0.3%	2.5%	93.4%	3.2%	1.4%	1.9%
18 months	21127	41.5%	55.2%	2.7%	0.6%	186	565	795	54.8%	43.2%	0.5%	0.1%	1.4%	94.1%	1.5%	0.8%	3.6%
2 years	19581	48.9%	48.6%	1.9%	0.6%	182	494	760	56.0%	42.2%	0.3%	0.1%	1.4%	94.0%	1.0%	0.4%	4.5%
3 years	18145	58.3%	39.9%	1.2%	0.6%	292	791	1493	56.2%	41.9%	0.2%	0.1%	1.6%	93.7%	0.7%	0.5%	5.1%
4 years	15569	64.2%	34.4%	1.0%	0.5%	252	737	1244	56.7%	41.2%	0.1%	0.1%	1.8%	93.3%	0.5%	0.5%	5.6%
5 years	13336	68.2%	30.5%	0.7%	0.6%	255	549	1278	57.8%	40.2%	0.1%	0.1%	1.9%	93.2%	0.4%	0.5%	5.9%
6 years	11254	70.4%	28.5%	0.6%	0.5%	207	503	1237	58.9%	39.2%	0.0%	0.0%	1.9%	92.3%	0.6%	0.4%	6.7%
7 years	9307	72.1%	27.0%	0.5%	0.4%	177	402	1158	58.9%	39.2%	0.0%	0.0%	1.8%	91.6%	0.6%	0.5%	7.2%
8 years	7570	73.4%	25.9%	0.4%	0.3%	143	310	1084	58.7%	38.8%	0.0%	0.1%	2.4%	90.5%	1.0%	0.5%	8.0%
9 years	6033	73.8%	25.4%	0.4%	0.3%	130	270	1025	59.8%	37.7%	0.1%	0.0%	2.3%	89.4%	0.7%	0.6%	9.3%
10 years	4608	74.1%	25.2%	0.4%	0.3%	95	203	883	59.2%	38.5%	0.0%	0.0%	2.3%	88.8%	0.9%	0.7%	9.6%

Figure S5. Trends in antiplatelet treatment in each sex group. Stacked bar graphs showing the relative proportion of the four different antiplatelet treatment in female patients (left panel). Proportion of antiplatelet agents prescribed for the patients who received mono-antiplatelet treatment (MAPT, middle panel) and those receiving dual-antiplatelet treatment (DAPT, right panel). asp = aspirin; clop = clopidogrel; tica = ticagrelor; pra = prasugrel; cilo = cilostazol.

A. Trends in antiplatelet treatment according to age groups: age of < 55.

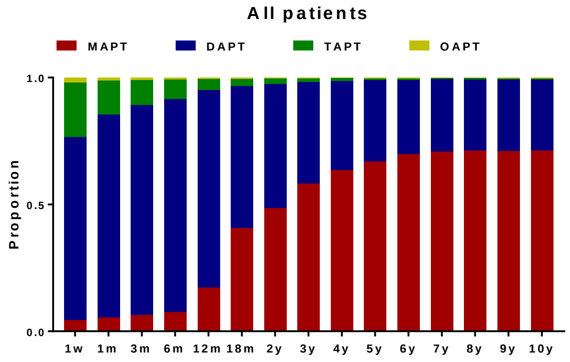

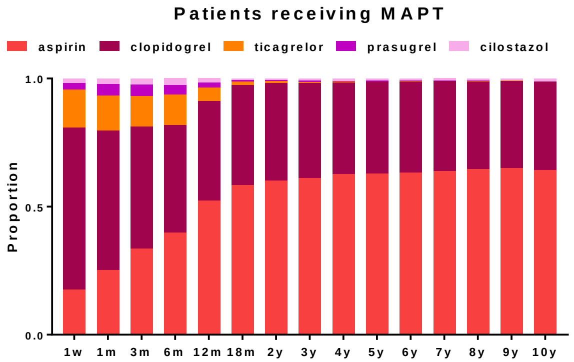

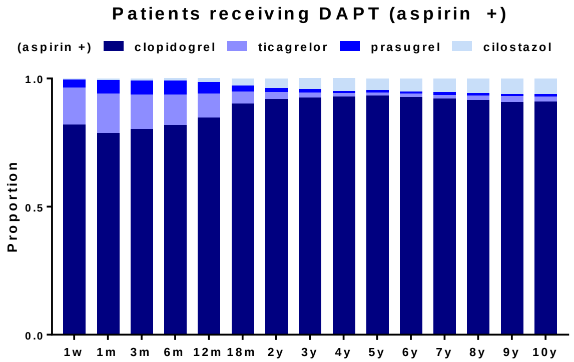

Date	Total	MAPT	DAPT	TAPT	OAPT	Death	No APT	Right-censored	MAPT					DAPT (aspirin +)			
	n					n	n	n	asp	clop	tica	pra	cilo	clop	tica	pra	cilo
1 week	19315	4.4%	72.2%	21.4%	2.0%	10	13	1	17.7%	63.1%	14.8%	2.7%	1.7%	82.0%	14.4%	3.2%	0.4%
1 month	19291	5.5%	80.0%	13.5%	1.1%	16	87	1	25.2%	54.3%	13.7%	4.6%	2.2%	78.8%	15.3%	5.4%	0.5%
3 months	19187	6.4%	82.7%	10.0%	0.9%	17	267	89	33.7%	47.6%	11.8%	4.4%	2.5%	80.2%	13.6%	5.4%	0.8%
6 months	18814	7.6%	83.9%	7.7%	0.8%	13	546	217	39.8%	42.0%	11.9%	3.8%	2.5%	81.8%	12.0%	5.3%	0.8%
12 months	18038	17.3%	77.7%	4.4%	0.6%	39	727	621	52.3%	38.8%	5.2%	2.1%	1.5%	84.7%	9.3%	4.5%	1.4%
18 months	16651	40.6%	56.0%	2.8%	0.5%	30	533	769	58.4%	39.1%	1.3%	0.7%	0.6%	90.2%	4.6%	2.3%	2.8%
2 years	15319	48.6%	48.9%	2.2%	0.4%	27	399	703	60.2%	38.0%	0.7%	0.6%	0.6%	92.0%	2.7%	1.6%	3.7%
3 years	14190	58.1%	40.1%	1.3%	0.4%	48	632	1382	61.1%	37.2%	0.4%	0.5%	0.8%	92.6%	1.9%	1.4%	4.1%
4 years	12128	63.5%	35.2%	1.0%	0.3%	41	507	1148	62.6%	35.9%	0.2%	0.3%	1.0%	92.9%	1.3%	0.9%	4.9%
5 years	10432	67.0%	31.9%	0.7%	0.3%	46	403	1142	63.0%	35.8%	0.1%	0.2%	0.9%	93.4%	1.2%	0.9%	4.6%
6 years	8841	69.9%	29.2%	0.6%	0.3%	43	344	1094	63.4%	35.4%	0.1%	0.2%	0.9%	92.8%	1.4%	0.8%	5.1%
7 years	7360	70.9%	28.4%	0.4%	0.3%	31	234	970	63.9%	35.0%	0.1%	0.1%	0.9%	92.2%	1.3%	1.1%	5.4%
8 years	6125	71.3%	28.0%	0.4%	0.3%	33	184	899	64.7%	34.2%	0.1%	0.2%	0.9%	91.5%	1.8%	0.9%	5.8%
9 years	5009	71.0%	28.2%	0.4%	0.4%	22	135	856	65.1%	33.9%	0.1%	0.0%	0.8%	90.9%	2.2%	1.0%	5.9%
10 years	3996	71.2%	28.0%	0.4%	0.4%	22	96	800	64.3%	34.4%	0.1%	0.0%	1.1%	91.1%	1.9%	1.0%	6.1%

B. Trends in antiplatelet treatment according to age groups: age of 55-64.

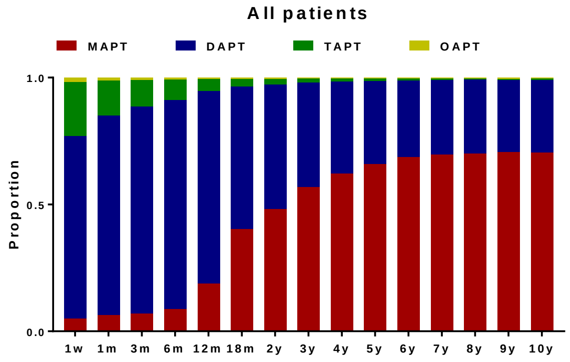

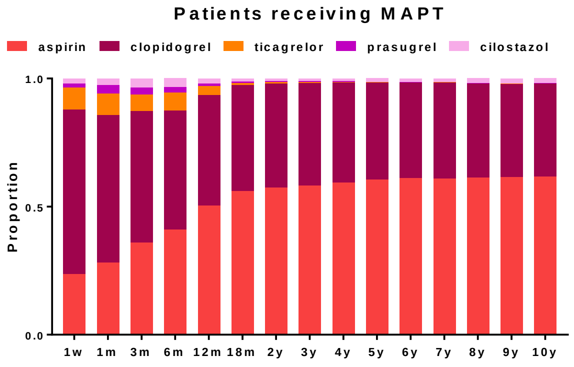

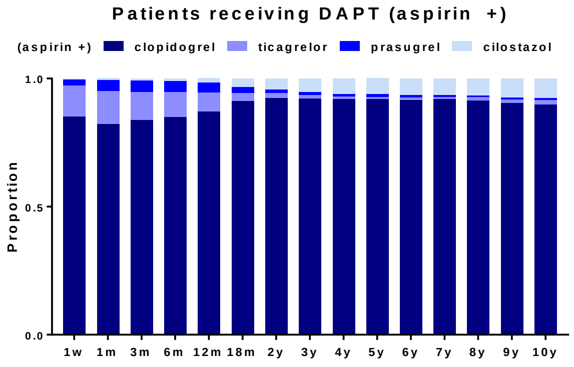

Date	Total	MAPT	DAPT	TAPT	OAPT	Death	No APT	Right-censored	MAPT					DAPT (aspirin +)			
	n					n	n	n	asp	clop	tica	pra	cilo	clop	tica	pra	cilo
1 week	23372	5.1%	71.8%	21.3%	1.8%	13	24	0	23.7%	64.0%	8.8%	1.5%	1.9%	85.2%	12.1%	2.3%	0.5%
1 month	23335	6.4%	78.5%	14.0%	1.1%	26	127	2	28.2%	57.5%	8.2%	3.5%	2.6%	82.3%	12.8%	4.3%	0.6%
3 months	23180	7.1%	81.5%	10.6%	0.9%	37	302	127	35.9%	51.4%	6.4%	2.8%	3.5%	83.7%	11.0%	4.5%	0.8%
6 months	22714	8.8%	82.4%	8.1%	0.8%	36	629	254	41.0%	46.3%	7.1%	2.3%	3.2%	85.0%	9.6%	4.4%	0.9%
12 months	21795	18.7%	76.0%	4.6%	0.7%	83	799	855	50.3%	43.3%	3.4%	1.1%	1.9%	87.1%	7.3%	3.9%	1.6%
18 months	20058	40.3%	56.1%	3.0%	0.6%	55	493	1037	56.1%	41.2%	0.9%	0.5%	1.3%	91.1%	3.3%	2.3%	3.3%
2 years	18473	48.1%	49.1%	2.2%	0.6%	50	401	959	57.5%	40.5%	0.6%	0.3%	1.1%	92.4%	1.8%	1.4%	4.4%
3 years	17063	57.0%	41.1%	1.5%	0.5%	118	589	1753	58.2%	40.0%	0.4%	0.3%	1.1%	92.3%	1.3%	1.2%	5.3%
4 years	14603	62.1%	36.3%	1.1%	0.5%	103	474	1516	59.4%	39.0%	0.2%	0.2%	1.1%	91.9%	0.9%	1.2%	6.0%
5 years	12510	65.9%	32.7%	0.8%	0.5%	88	372	1389	60.5%	38.0%	0.1%	0.2%	1.3%	92.0%	0.8%	1.0%	6.2%
6 years	10661	68.7%	30.1%	0.8%	0.5%	86	365	1319	61.2%	37.1%	0.1%	0.2%	1.4%	91.5%	1.1%	0.9%	6.5%
7 years	8891	69.7%	29.3%	0.6%	0.5%	85	254	1255	61.0%	37.4%	0.1%	0.0%	1.4%	92.0%	0.8%	0.8%	6.5%
8 years	7297	70.0%	29.1%	0.5%	0.3%	77	197	1167	61.3%	36.6%	0.1%	0.0%	1.9%	91.3%	1.5%	0.5%	6.7%
9 years	5856	70.6%	28.3%	0.5%	0.5%	62	167	1023	61.6%	36.3%	0.1%	0.0%	2.0%	90.4%	1.3%	0.8%	7.5%
10 years	4604	70.4%	28.6%	0.6%	0.5%	61	137	886	61.7%	36.3%	0.1%	0.0%	1.9%	89.7%	1.9%	0.6%	7.8%

C. Trends in antiplatelet treatment according to age groups: age of 65-74.

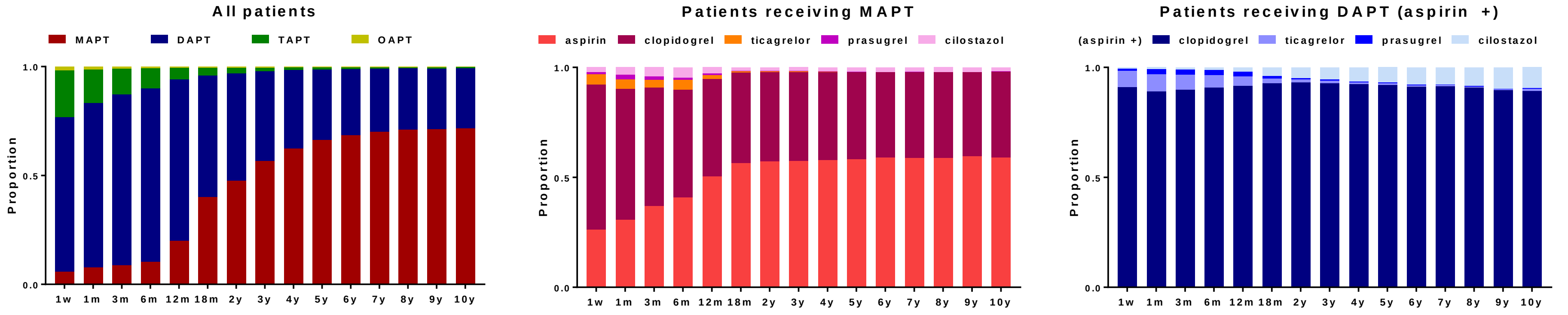

Date	Total	MAPT	DAPT	TAPT	OAPT	Death	No APT	Right-censored	MAPT					DAPT (aspirin +)			
	n					n	n	n	asp	clop	tica	pra	cilo	clop	tica	pra	cilo
1 week	23094	5.7%	71.0%	21.6%	1.7%	30	22	1	26.1%	66.1%	4.6%	1.1%	2.1%	91.1%	7.3%	1.1%	0.5%
1 month	23041	7.7%	75.5%	15.5%	1.3%	51	107	5	30.8%	59.5%	4.2%	2.3%	3.3%	88.9%	7.9%	2.4%	0.8%
3 months	22878	8.7%	78.6%	11.7%	1.0%	87	347	91	36.9%	54.0%	3.3%	1.7%	4.1%	89.9%	6.7%	2.4%	1.0%
6 months	22353	10.3%	79.8%	9.1%	0.9%	95	703	211	40.7%	49.2%	4.3%	1.2%	4.6%	90.8%	5.6%	2.3%	1.2%
12 months	21344	19.9%	74.2%	5.2%	0.7%	220	789	661	50.5%	44.2%	1.9%	0.6%	2.8%	91.5%	4.4%	2.0%	2.1%
18 months	19674	40.0%	55.9%	3.4%	0.7%	167	519	723	56.4%	41.2%	0.6%	0.3%	1.5%	92.8%	2.1%	1.2%	3.9%
2 years	18265	47.5%	49.4%	2.4%	0.6%	144	374	685	57.1%	40.8%	0.3%	0.2%	1.5%	93.1%	1.4%	0.7%	4.8%
3 years	17062	56.6%	41.2%	1.6%	0.6%	272	668	1382	57.4%	40.6%	0.3%	0.1%	1.6%	92.8%	1.0%	0.6%	5.6%
4 years	14740	62.4%	36.1%	1.1%	0.5%	245	570	1233	57.9%	39.9%	0.2%	0.1%	1.9%	92.4%	0.6%	0.5%	6.4%
5 years	12692	66.3%	32.4%	0.9%	0.5%	244	481	1187	58.3%	39.4%	0.1%	0.1%	2.0%	92.0%	0.6%	0.4%	6.9%
6 years	10780	68.4%	30.4%	0.7%	0.5%	226	416	1105	59.0%	38.7%	0.1%	0.1%	2.1%	91.2%	0.6%	0.3%	7.9%
7 years	9033	70.2%	28.9%	0.6%	0.4%	197	370	1179	58.7%	39.2%	0.0%	0.0%	2.0%	91.4%	0.5%	0.2%	7.9%
8 years	7287	71.1%	28.0%	0.5%	0.3%	178	293	1022	58.8%	38.9%	0.1%	0.1%	2.2%	90.8%	0.5%	0.3%	8.4%
9 years	5794	71.3%	27.8%	0.5%	0.4%	159	245	1026	59.5%	38.3%	0.1%	0.0%	2.1%	89.7%	0.3%	0.2%	9.7%
10 years	4364	71.6%	27.7%	0.4%	0.3%	111	203	824	59.0%	39.0%	0.1%	0.0%	1.9%	89.2%	1.1%	0.2%	9.4%

D. Trends in antiplatelet treatment according to age groups: age of ≥75.

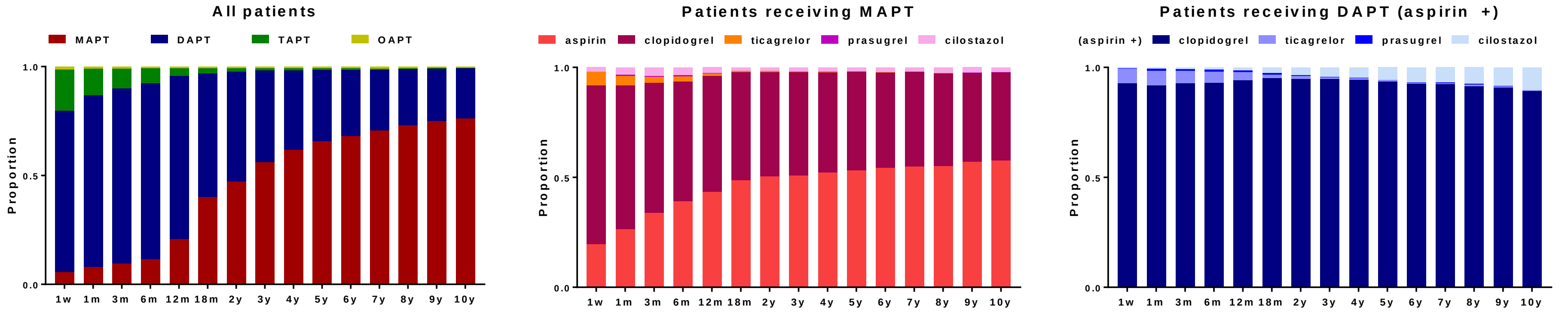

Date	Total	MAPT	DAPT	TAPT	OAPT	Death	No APT	Right-censored	MAPT					DAPT (aspirin +)			
	n					n	n	n	asp	clop	tica	pra	cilo	clop	tica	pra	cilo
1 week	13873	5.6%	74.2%	18.9%	1.4%	37	24	0	19.5%	72.3%	6.2%	0.0%	1.9%	92.7%	6.7%	0.3%	0.3%
1 month	13812	8.0%	78.7%	12.2%	1.0%	77	103	2	26.3%	65.5%	4.2%	0.5%	3.4%	91.8%	6.7%	0.9%	0.7%
3 months	13630	9.5%	80.3%	9.2%	1.0%	173	410	79	33.8%	59.1%	2.7%	0.5%	3.9%	92.7%	5.7%	0.9%	0.7%
6 months	12968	11.5%	80.8%	6.7%	0.9%	196	614	157	39.2%	54.3%	2.4%	0.4%	3.7%	93.0%	5.0%	1.0%	1.1%
12 months	12001	20.9%	74.8%	3.4%	0.9%	305	647	476	43.3%	52.7%	1.2%	0.1%	2.7%	94.0%	3.8%	0.8%	1.4%
18 months	10573	40.2%	56.7%	2.4%	0.7%	236	377	535	48.6%	49.2%	0.6%	0.0%	1.6%	95.0%	1.7%	0.6%	2.6%
2 years	9425	47.2%	50.4%	1.8%	0.7%	217	335	524	50.4%	47.5%	0.4%	0.0%	1.6%	94.7%	1.3%	0.4%	3.6%
3 years	8349	56.1%	42.0%	1.2%	0.7%	367	471	913	50.7%	47.2%	0.3%	0.1%	1.8%	94.7%	0.8%	0.3%	4.2%
4 years	6598	61.8%	36.4%	1.0%	0.8%	293	406	699	52.1%	45.6%	0.2%	0.0%	2.0%	94.3%	0.7%	0.2%	4.7%
5 years	5200	65.7%	32.7%	0.7%	0.8%	246	298	667	53.2%	44.7%	0.2%	0.0%	1.8%	93.6%	0.6%	0.2%	5.6%
6 years	3989	68.1%	30.3%	0.6%	0.9%	210	252	569	54.4%	43.2%	0.1%	0.0%	2.2%	92.6%	0.3%	0.2%	6.9%
7 years	2958	70.6%	28.0%	0.5%	0.9%	170	189	450	55.0%	42.9%	0.0%	0.0%	2.1%	92.4%	0.4%	0.4%	6.9%
8 years	2149	73.0%	25.8%	0.3%	0.8%	123	130	366	55.0%	42.3%	0.1%	0.1%	2.5%	91.4%	0.9%	0.4%	7.4%
9 years	1530	74.9%	24.1%	0.5%	0.6%	102	102	318	57.1%	40.3%	0.1%	0.1%	2.4%	90.8%	0.5%	0.3%	8.4%
10 years	1008	76.2%	22.9%	0.4%	0.5%	62	73	221	57.7%	40.0%	0.0%	0.1%	2.2%	89.2%	0.4%	0.0%	10.4%

Figure S6. Trends in antiplatelet treatment in each age group. Stacked bar graphs showing the relative proportion of the four different antiplatelet treatment in patients with age of age of ≥75 (left panel). Proportion of antiplatelet agents prescribed for the patients who received mono-antiplatelet treatment (MAPT, middle panel) and those receiving dual-antiplatelet treatment (DAPT, right panel). asp = aspirin; clop = clopidogrel; tica = ticagrelor; pra = prasugrel; cilo = cilostazol

A. Trends in antiplatelet treatment in AMI patients.

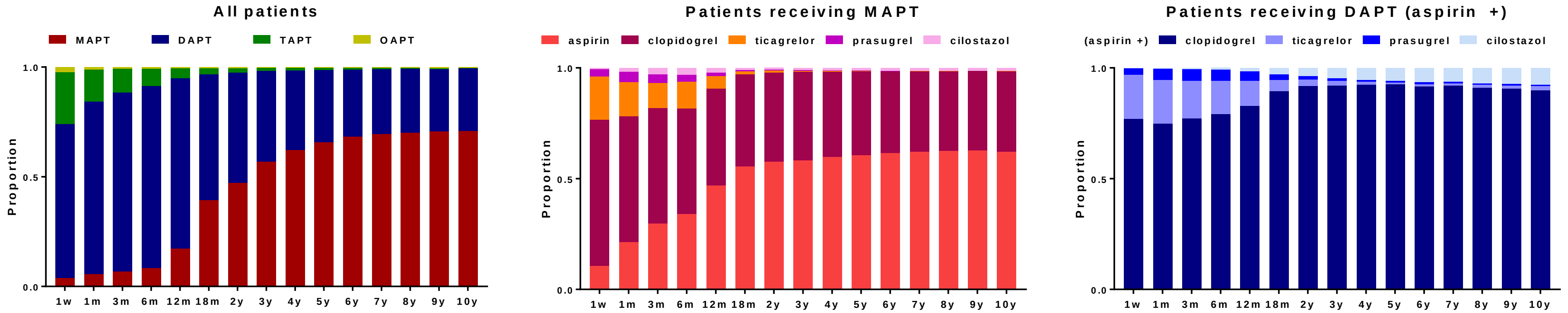

Date	Total	MAPT	DAPT	TAPT	OAPT	Death	No APT	Right-censored	MAPT					DAPT (aspirin +)			
	n					n	n	n	asp	clop	tica	pra	cilo	clop	tica	pra	cilo
1 week	30823	3.9%	70.0%	23.8%	2.3%	69	29	1	10.5%	65.9%	19.6%	3.3%	0.7%	76.9%	19.9%	3.1%	0.2%
1 month	30724	5.5%	78.8%	14.5%	1.2%	107	158	4	21.4%	56.6%	15.6%	4.7%	1.8%	74.8%	19.8%	5.0%	0.4%
3 months	30455	6.8%	81.7%	10.6%	0.9%	176	522	163	29.7%	52.1%	11.3%	3.8%	3.1%	77.1%	17.1%	5.1%	0.8%
6 months	29594	8.3%	83.0%	7.9%	0.8%	147	854	364	34.1%	47.5%	12.1%	3.2%	3.1%	79.1%	15.0%	5.1%	0.8%
12 months	28229	17.1%	77.8%	4.4%	0.7%	294	1141	1107	46.9%	43.8%	5.6%	1.6%	2.2%	82.8%	11.3%	4.4%	1.5%
18 months	25687	39.4%	57.2%	2.8%	0.6%	216	824	1303	55.4%	41.5%	1.5%	0.5%	1.0%	89.4%	5.2%	2.5%	3.0%
2 years	23344	47.2%	50.1%	2.1%	0.5%	184	593	1196	57.5%	40.4%	0.8%	0.4%	0.9%	91.7%	3.0%	1.5%	3.8%
3 years	21371	56.8%	41.3%	1.4%	0.5%	347	888	2240	58.2%	39.9%	0.5%	0.3%	1.1%	92.1%	2.1%	1.2%	4.6%
4 years	17896	62.1%	36.3%	1.0%	0.5%	303	762	1904	59.9%	38.3%	0.3%	0.3%	1.2%	92.4%	1.3%	0.9%	5.4%
5 years	14927	65.7%	33.0%	0.9%	0.5%	271	585	1783	60.5%	37.9%	0.2%	0.2%	1.2%	92.5%	1.0%	0.8%	5.8%
6 years	12288	68.3%	30.6%	0.7%	0.4%	209	509	1629	61.5%	36.7%	0.1%	0.2%	1.4%	91.6%	1.0%	0.8%	6.6%
7 years	9941	69.4%	29.6%	0.5%	0.4%	206	348	1429	62.1%	36.4%	0.1%	0.0%	1.3%	92.0%	0.9%	0.7%	6.3%
8 years	7958	70.1%	29.0%	0.6%	0.4%	167	291	1212	62.5%	35.9%	0.1%	0.1%	1.4%	91.0%	1.3%	0.6%	7.1%
9 years	6288	70.6%	28.3%	0.5%	0.6%	141	212	1060	62.7%	35.8%	0.1%	0.0%	1.4%	90.6%	1.3%	0.7%	7.4%
10 years	4875	70.9%	28.4%	0.3%	0.3%	99	177	929	62.0%	36.3%	0.1%	0.1%	1.4%	89.8%	1.9%	0.4%	7.8%

B. Trends in antiplatelet treatment in non-AMI patients.

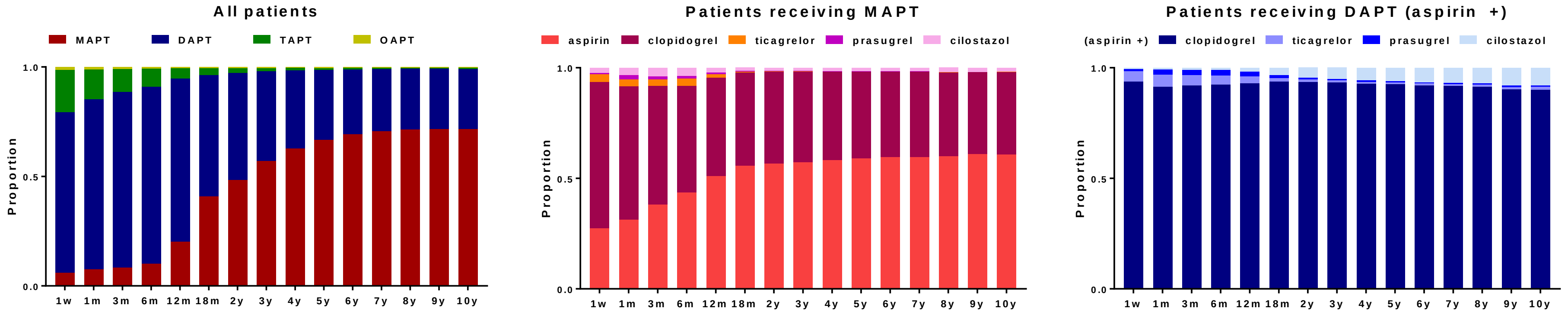

Date	Total	MAPT	DAPT	TAPT	OAPT	Death	No APT	Right-censored	MAPT					DAPT (aspirin +)			
	n					n	n	n	asp	clop	tica	pra	cilo	clop	tica	pra	cilo
1 week	48831	5.9%	73.4%	19.2%	1.4%	21	54	1	27.4%	66.1%	3.5%	0.5%	2.5%	93.8%	4.5%	1.1%	0.6%
1 month	48755	7.7%	77.5%	13.7%	1.1%	63	266	6	31.2%	60.3%	3.3%	1.8%	3.4%	91.4%	5.3%	2.4%	0.8%
3 months	48420	8.4%	80.2%	10.4%	1.0%	138	804	223	38.2%	53.6%	2.9%	1.5%	3.8%	91.9%	4.7%	2.5%	0.9%
6 months	47255	10.1%	80.9%	8.1%	0.9%	193	1638	475	43.5%	48.3%	3.2%	1.2%	3.9%	92.4%	4.0%	2.5%	1.1%
12 months	44949	20.3%	74.4%	4.6%	0.7%	353	1821	1506	51.0%	44.5%	1.5%	0.7%	2.3%	93.0%	3.1%	2.1%	1.8%
18 months	41269	40.9%	55.4%	3.1%	0.7%	272	1098	1761	55.6%	42.2%	0.5%	0.3%	1.4%	93.7%	1.6%	1.3%	3.4%
2 years	38138	48.3%	48.9%	2.2%	0.6%	254	916	1675	56.7%	41.4%	0.3%	0.2%	1.4%	93.6%	1.1%	0.8%	4.5%
3 years	35293	57.1%	40.8%	1.5%	0.6%	458	1472	3190	57.2%	40.9%	0.3%	0.2%	1.4%	93.4%	0.8%	0.7%	5.1%
4 years	30173	62.7%	35.7%	1.1%	0.5%	379	1195	2692	58.1%	40.0%	0.2%	0.1%	1.6%	92.8%	0.7%	0.7%	5.8%
5 years	25907	66.6%	32.1%	0.8%	0.5%	353	969	2602	59.0%	39.1%	0.1%	0.1%	1.6%	92.6%	0.7%	0.6%	6.0%
6 years	21983	69.2%	29.6%	0.7%	0.5%	356	868	2458	59.6%	38.5%	0.1%	0.1%	1.7%	92.0%	0.9%	0.5%	6.6%
7 years	18301	70.7%	28.3%	0.5%	0.5%	277	699	2425	59.5%	38.7%	0.1%	0.1%	1.6%	91.8%	0.7%	0.6%	6.9%
8 years	14900	71.5%	27.7%	0.4%	0.4%	244	513	2242	60.0%	37.9%	0.1%	0.1%	2.0%	91.3%	1.1%	0.5%	7.0%
9 years	11901	71.6%	27.5%	0.5%	0.4%	204	437	2163	60.9%	37.1%	0.1%	0.0%	1.9%	90.2%	1.1%	0.6%	8.1%
10 years	9097	71.7%	27.3%	0.5%	0.4%	157	332	1802	60.9%	37.2%	0.1%	0.0%	1.9%	90.0%	1.3%	0.6%	8.0%

Figure S7. Trends in antiplatelet treatment in AMI and non-AMI patients. Stacked bar graphs showing the relative proportion of the four different antiplatelet treatment in non-AMI patients (left panel). Proportion of antiplatelet agents prescribed for the patients who received mono-antiplatelet treatment (MAPT, middle panel) and those receiving dual-antiplatelet treatment (DAPT, right panel). asp = aspirin; clop = clopidogrel; tica = ticagrelor; pra = prasugrel; cilo = cilostazol
